# Gasdermin D‐Mediated Release of IL‐33 Results in Fetal Brain Developmental Abnormalities During Maternal Colitis

**DOI:** 10.1002/advs.202523784

**Published:** 2026-04-13

**Authors:** Huiyang Jia, Shukui Zhang, Kai Ma, Jie Zhou, Jianwei Jiao

**Affiliations:** ^1^ State Key Laboratory of Organ Regeneration and Reconstruction Institute of Zoology Chinese Academy of Sciences Beijing China; ^2^ University of Chinese Academy of Sciences Beijing China; ^3^ Beijing Institute for Stem Cell and Regenerative Medicine Beijing China; ^4^ Co‐Innovation Center of Neuroregeneration Nantong University Nantong China

**Keywords:** brain development, colitis, Gasdermin D, IL‐33, neural stem cells

## Abstract

Maternal inflammatory bowel disease (IBD) is associated with an increased incidence of autism spectrum disorder in offspring. The mechanism behind this phenomenon remains largely unknown. Here, we found that offspring from colitic dams exhibit increased brain weight. These offspring also show excessive neural stem cell (NSC) proliferation and behavioral deficits. Furthermore, Gsdmd cleavage is elevated in the colonic epithelium of colitic dams. This increase is associated with developmental abnormalities in the fetal brain. Mechanistically, excessive NSC proliferation is driven by increased IL‐33 release from the maternal colonic epithelium in a Gsdmd‐dependent manner. In contrast, we found no evidence that maternal luminal LPS leakage or increased fetal microglia accounts for this phenotype. Our study demonstrates that excessive pyroptosis in the maternal colonic epithelium leads to neurodevelopmental defects and disrupts neuroimmune homeostasis in the offspring.

## Graphical abstract

Under colitis, Gsdmd mediates the release of IL‐33 from the epithelium of pregnant mice. IL‐33 can cross the placenta and enhance the proliferative capacity of neural stem cells, ultimately resulting in behavioral deficits in the offspring. Excessive pyroptosis in the colonic epithelium also triggers the translocation of LPS, which in turn increases the number of microglia in the fetal brain.

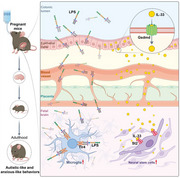

## 1 Introduction

Approximately 27% of patients with IBD develop extraintestinal symptoms [1]. A 44% increased risk of autism has been observed in children of mothers with ulcerative colitis (UC) [2]. Although the clinical association between UC and the occurrence of autism in offspring has been established, the underlying molecular mechanisms remain unclear.

Maternal immune activation is closely associated with autism‐related phenotypes in offspring [3, 4, 5, 6]. Previous studies have shown that IL‐17 and granzyme B produced by maternal mice after simulated viral infection can cross the placenta and further affect fetal neurogenesis [4] and neuroimmune homeostasis [3].

UC patients present with continuous colonic mucosal inflammation, accompanied by elevated cytokine secretion levels [7, 8]. The gasdermin family mediates the progression of pyroptosis‐associated inflammation and the release of proinflammatory cytokines [9, 10, 11]. Knocking out Gsdmd in cerebrovascular endothelial cells prevents LPS‐induced disruption of the blood‐brain barrier [12], while the use of Gsdmd pore‐forming inhibitors blocks the excessive release of proinflammatory cytokines and improves the prognosis of mice [13, 14].

We established a pregnant mouse model of colitis using dextran sulfate sodium (DSS). We found that fetuses from colitic dams exhibited increased proliferative capacity of NSCs; furthermore, these offspring displayed autism‐like and anxiety‐like behaviors in postnatal behavioral tests. Under colitis conditions, more cleavage of Gsdmd was observed in the colonic epithelium compared with Gsdma, Gsdmc, and Gsdme. Conditional knockout of Gsdmd in the colonic epithelium of maternal mice rescued abnormal brain development and behavioral defects in offspring mice. Through RNA sequencing (RNA‐seq) analysis of the colonic epithelium and receptor knockdown experiments in the fetal mouse brain, we identified interleukin‐33 (IL‐33) released via Gsdmd as a key factor driving abnormal proliferation of NSCs. Meanwhile, during colitis, we observed that LPS derived from the maternal gut could cross the placenta and translocate into the fetal mouse brain, leading to an increase in microglia within the fetal brain. However, knockout of *Toll‐like receptor 4* (*Tlr4*) failed to rescue the excessive proliferation of NSCs. Additionally, depletion of microglia using PLX5622 (colony‐stimulating factor 1 receptor inhibitor) or by transgenic approaches also failed to ameliorate the abnormal proliferation of NSCs.

## 2 Results

### 2.1 Maternal Colitis Is Associated with Reduced Body Weight and Increased Brain Weight in Offspring

**FIGURE 1:**
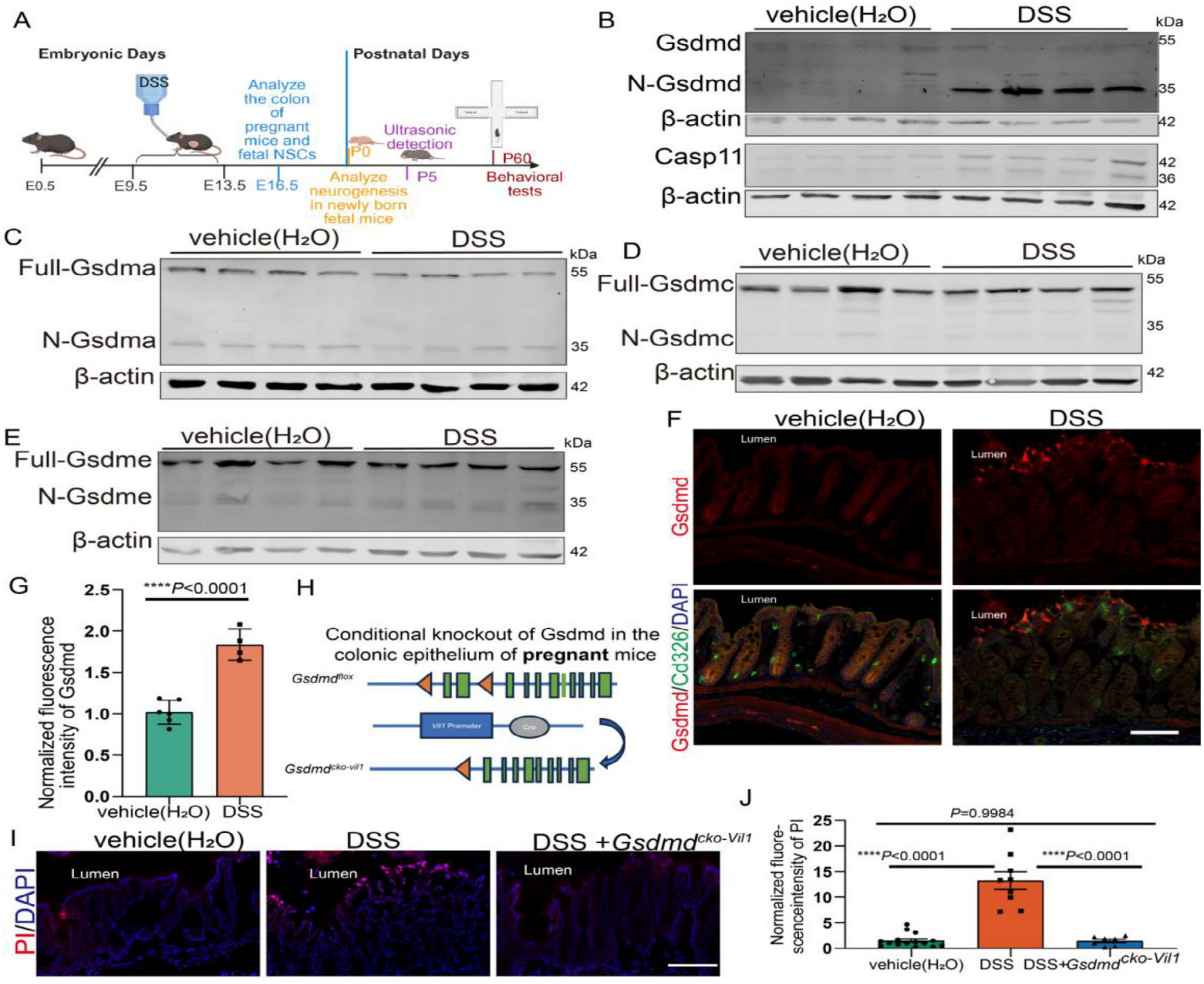
Gsdmd mediates pyroptosis of colonic epithelial cells in colitis. (A) Flowchart of DSS‐induced colitis modeling and subsequent experiments. Created with BioRender.com. (B–E) Gsdmd, Gsdma, Gsdmc, and Gsdme expression in colonic epithelium of DSS‐treated or control dams (*n* = 4 per group). (F,G) Immunofluorescence showing the localization (F) and relative expression level of Gsdmd in colon (G). (*n* = 6, vehicle (H_2_O) group; *n* = 4, DSS group). Scale bar = 100 µm. (H) Method for constructing Gsdmd conditional knockout mice. (I,J) Representative image of PI‐treated colon (I). PI staining intensity of each group (J). (*n* = 15, vehicle (H_2_O) group; *n* = 9, DSS group; *n* = 7, DSS + *Gsdmd^cko‐Vil1^* group). Scale bar = 100 µm. Each data point in the statistical plots represents one biological replicate, and each experiment was performed with three technical replicates. Data were analyzed with an unpaired two‐tailed *t*‐test (G), and with one‐way ANOVA followed by Tukey's multiple comparisons test (J). All data are presented as the mean ± SEM.

To investigate whether maternal colitis induces offspring abnormalities, we treated pregnant mice with 2.5% DSS from embryonic day 9.5 (E9.5) to E13.5 (Figure 1A). At E16.5, pathological analysis confirmed that the pregnant mice remained in a state of colitis, as evidenced by increased colonic pathological scores and loss of colonic apical epithelial cells (Figure S1A–C). We monitored the body weight of the pregnant mice and found that, compared with the vehicle (H_2_O) group, the DSS‐treated pregnant mice gained weight more slowly (Figure S1D). After the colitic dams gave birth, we found that the newborn mice from DSS‐treated pregnant mice exhibited decreased body weight; intriguingly, their brain weight was increased (Figure S1E–G).

### 2.2 Gsdmd Mediates Pyroptosis of Colonic Epithelial Cells in Colitis

The Gasdermin family mediates pyroptosis‐associated inflammation [9, 13]. To clarify the activation of Gasdermin family molecules during colitis, we examined the expression and cleavage of Gsdma, Gsdmc, Gsdmd, and Gsdme in colonic epithelial cells. We found that Gsdmd was the most prominently cleaved molecule (Figure 1B–E). Furthermore, through analysis of single‐cell transcriptome data from human UC, we observed increased GSDMD expression in multiple types of intestinal epithelial cells (Figure S2A); elevated pyroptosis‐related genes were also detected in transcriptome sequencing of colitis tissues from mice (Figure S2B). By immunofluorescence staining of colonic tissue, we found that under colitis conditions, Gsdmd expression was primarily localized in the apical mucosal epithelium (Figure 1F,G). Next, we generated colonic epithelial‐specific Gsdmd knockout mice and verified the knockout efficiency (Figure 1H; Figure S1H). We performed intraluminal injection of Propidium Iodide (PI) dye; our results showed that the abnormal pyroptosis in colonic epithelium could be rescued by Gsdmd knockout (Figure 1I,J). In addition, we detected no difference in colonic epithelial cell death between Gsdmd‑knockout and wild‑type mice under normal drinking water conditions (Figure S1I,J). We found that although the knockout of Gsdmd in colonic epithelial cells could not completely reverse the pathological score of colitis, it can maintain the number of apical colonic epithelial cells at a normal density (Figure S1A–C).

### 2.3 Behavioral Abnormalities in Offspring Induced by Maternal Colitis Can be Rescued by Knocking Out Gsdmd in the Maternal Colonic Epithelium

**FIGURE 2:**
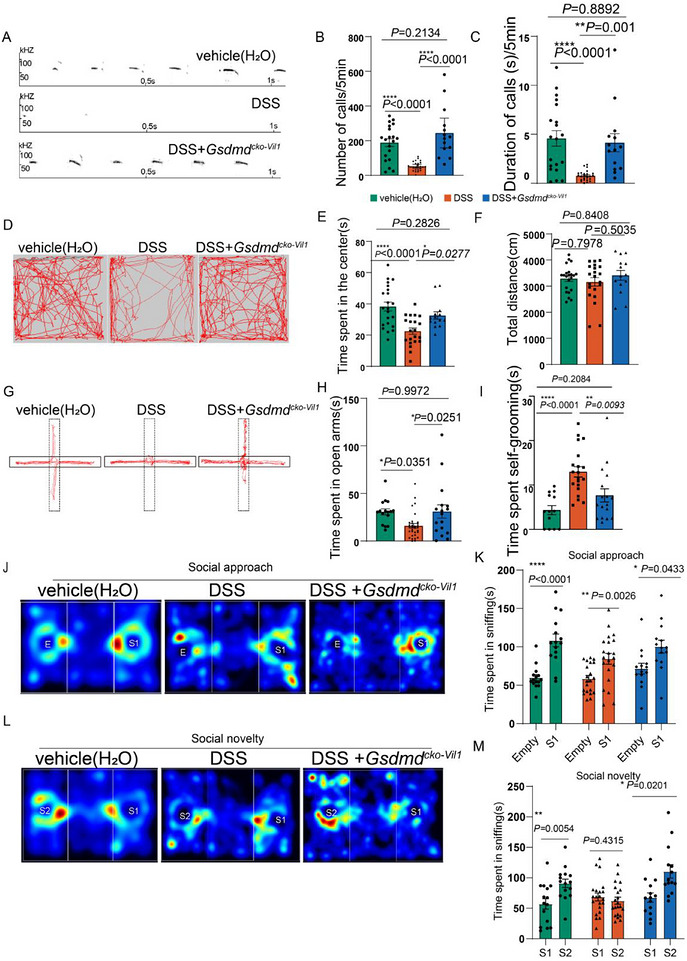
Behavioral deficits in offspring induced by maternal colitis can be rescued by knocking out Gsdmd in the maternal colonic epithelium. (A–C) Representative ultrasonic vocalizations (USVs) spectrograms at P5 (A). Statistics on the number (B) and total duration (C) of ultrasonic vocalizations within 5 min. (*n* = 20, vehicle (H_2_O) group; *n* = 25, DSS group; *n* = 14, DSS + *Gsdmd^cko‐Vil1^* group). (D–F) Representative track plots of the open field test (D). Duration of exploration in the central area (E) and total distance in the open field (F). (*n* = 22, vehicle (H_2_O) group; *n* = 21, DSS group; *n* = 14, DSS + *Gsdmd^cko‐Vil1^* group). (G,H) Representative track plots of the elevated plus maze test (G). Total time spent in open arms (H). (*n* = 16, vehicle (H_2_O) group; *n* = 32, DSS group; *n* = 17, DSS + *Gsdmd^cko‐Vil1^* group). (I) Duration of stereotypic grooming behavior in mice within 5 min. (*n* = 13, vehicle (H_2_O) group; *n* = 20, DSS group; *n* = 17, DSS + *Gsdmd^cko‐Vil1^* group). (J–M) Representative track heatmaps for social approach and social novelty (J and L). Comparison of the durations of mice exploring Stranger 1 and the empty cage (K). Comparison of the durations of mice exploring Stranger 1 and Stranger 2 (M). (*n* = 15, vehicle (H_2_O) group; *n* = 21, DSS group; *n* = 14, DSS + *Gsdmd^cko‐Vil1^* group). Each data point in the statistical plots represents one biological replicate, and each experiment was performed with one technical replicate. Data were analyzed with unpaired two‐tailed *t*‐tests (K and M), and with one‐way ANOVA followed by Tukey's multiple comparisons test (B, C, E, F, H, and I). All data are presented as the mean ± SEM.

To determine whether offspring of colitic dams truly exhibit autism‐like behaviors, we conducted a series of behavioral tests. First, we performed ultrasonic vocalization tests on neonatal mice. We found that, compared with the DSS‐treated group, both the vehicle (H_2_O) group and the DSS + *Gsdmd^cko‐Vil1^* group showed longer duration and frequency of ultrasonic vocalizations, and there was no significant difference in the duration and frequency of ultrasonic vocalizations between the vehicle (H_2_O) group and the DSS + *Gsdmd^cko‐Vil1^* group (Figure 2A–C). This suggests that the offspring of colitis mice have impairments in communication. After the mice reached adulthood (postnatal day 60), we continued the behavioral tests. We first conducted the open field test on the mice and found that DSS treatment did not lead to a reduction in the mice's locomotor ability; however, the time the mice spent in the central area of the field decreased following DSS treatment during pregnancy. Knocking out Gsdmd in the maternal colonic epithelium reversed this phenomenon (Figure 2D–F). In the elevated plus maze test, mice in the DSS‐treated group spent less time exploring the open arms, while knocking out Gsdmd in the colonic epithelium of maternal mice restored the exploration time to the level of the vehicle (H_2_O) group (Figure 2G,H). Furthermore, we also observed that knocking out Gsdmd in the colonic epithelium of maternal mice could rescue the increase in stereotypical behaviors in offspring induced by maternal colitis during pregnancy, as determined by observing the grooming behavior of the mice (Figure 2I). In the three‐chamber social test, mice in all groups preferred to stay in and sniff the stranger mouse rather than the empty cage. However, in the social novelty test, the offspring of DSS‐treated dams spent equal time sniffing Strangers 1 and 2, whereas the vehicle (H_2_O) group and the DSS + *Gsdmd^cko‐Vil1^* group showed greater interest in Stranger 2 (Figure 2J–M). This suggests that DSS‐treated offspring exhibit impaired social memory.

We reanalyzed the behavioral differences between male and female offspring mice in the DSS‐treated group and found that there were no statistically significant differences between males and females in all the aforementioned behavioral tests (Figure S3A–I). Therefore, both male and female mice were included in the statistical analysis of subsequent mechanism‐related experiments.

We conducted the forced swim test and novel object recognition test at the end. We found that in the forced swim test, there was no significant difference in immobility time between the offspring of the vehicle (H_2_O) group and the DSS‐treated group (Figure S3J). In the novel object recognition test, the offspring of the vehicle (H_2_O) group and the colitis group also showed no significant difference in the time spent exploring the novel object (Figure S3K). This indicates that colitis does not induce depressive‐like behaviors or cognitive deficits in offspring.

### 2.4 Excessive Proliferation and Neurogenesis of Fetal NSCs in Offspring of Colitic Dams Can be Rescued by Knockout of Gsdmd in the Maternal Colonic Epithelium

**FIGURE 3:**
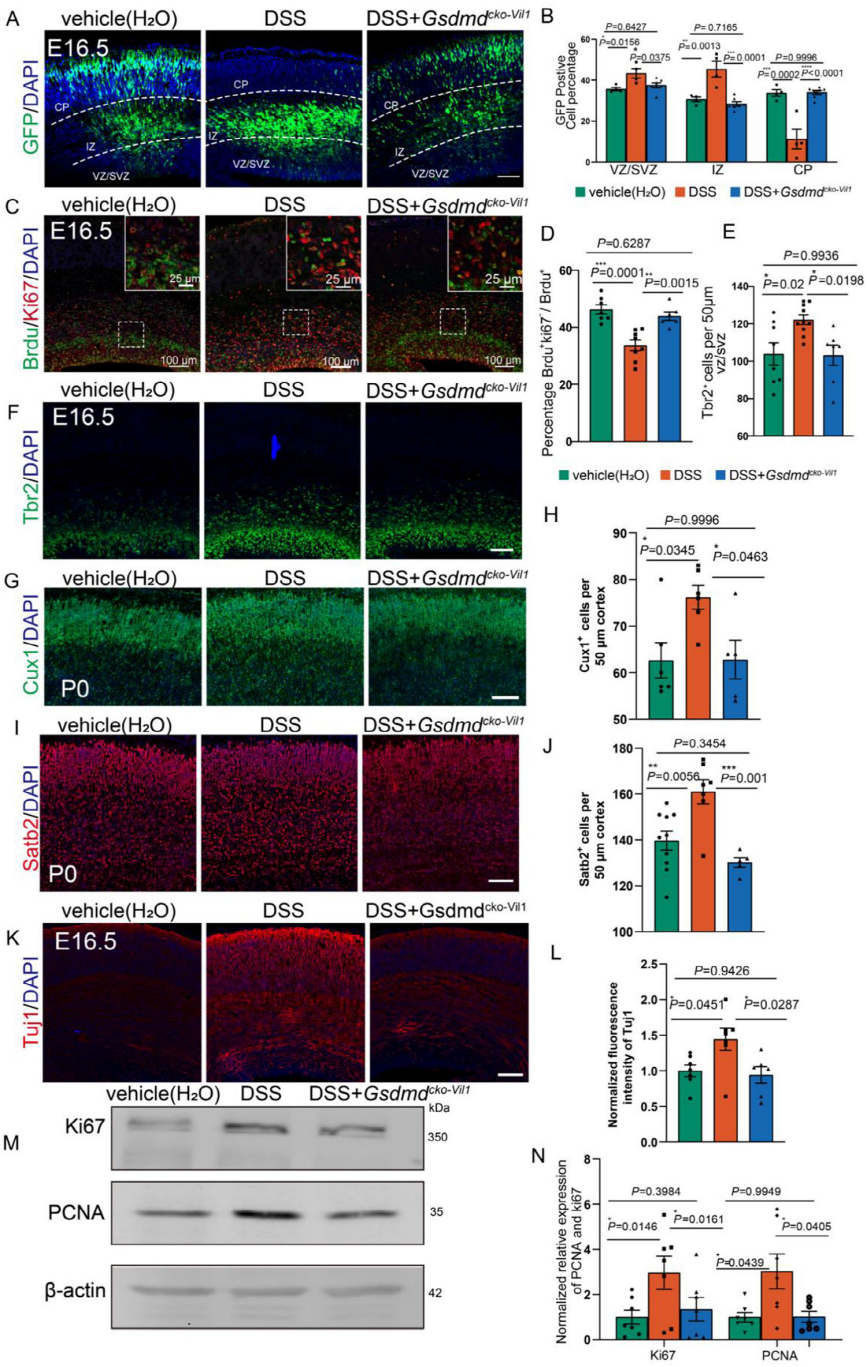
Excessive proliferation and neurogenesis of fetal NSCs in offspring of colitic dams can be rescued by knockout of Gsdmd in the maternal colonic epithelium. (A) GFP plasmids were electroporated into the brains of E13.5 offspring and analyzed at E16.5. Scale bar = 100 µm. (B) The bar graph shows the quantitative analysis of the percentage of GFP‐positive cells in different regions of the cerebral cortex. (*n* = 4, vehicle (H_2_O) group; *n* = 4, DSS group; *n* = 7, DSS + *Gsdmd^cko‐Vil1^* group). (R^2^ = 0.507; 95% confidence interval of the difference: vehicle (H_2_O) vs DSS: ‐0.1398 to ‐0.015; vehicle (H_2_O) vs DSS + *Gsdmd^cko‐Vil1^*: ‐0.073 to 0.036; DSS vs DSS + *Gsdmd^cko‐Vil1^*: 0.003 to 0.113). (C) The status of cell cycle exit (BrdU^+^ Ki67^−^) 24 h after BrdU injection in E16.5 cortex. (D) Analysis of the proportion of cell cycle exit in the three groups of mice. (*n* = 7, vehicle (H_2_O) group; *n* = 8, DSS group; *n* = 6, DSS + *Gsdmd^cko‐Vil1^* group). (E,F) Representative immunofluorescence images of Tbr2^+^ cells in E16.5 cerebral cortex for each group (F), and bar graph for the statistical analysis of Tbr2^+^ cell number (E). (*n* = 8, vehicle (H_2_O) group; *n* = 10, DSS group; *n* = 7, DSS + *Gsdmd^cko‐Vil1^* group). Scale bar = 100 µm. (G,H) Representative immunofluorescence images of Cux1^+^ cells at P0 (G), and statistical analysis of the number of Cux1^+^ cells (H). (*n* = 6, vehicle (H_2_O) group; *n* = 6, DSS group; *n* = 5, DSS + *Gsdmd^cko‐Vil1^* group). Scale bar = 100 µm. (I,J) Representative immunofluorescence images of Satb2^+^ cells at P0 (I), and statistical analysis of the number of Satb2^+^ cells in three groups of mice (J). (*n* = 10, vehicle (H_2_O) group; *n* = 7, DSS group; *n* = 5, DSS + *Gsdmd^cko‐Vil1^* group). Scale bar = 100 µm. (K,L) Representative immunofluorescence images of Tuj1 at E16.5 (K), and statistical analysis of the intensity of Tuj1 in three groups of mice (L). (*n* = 7, vehicle (H_2_O) group; *n* = 7, DSS group; *n* = 6, DSS + *Gsdmd^cko‐Vil1^* group). Scale bar = 100 µm. (M,N) Western blot analysis of the expression levels of Ki67 and PCNA in the E16.5 cerebral cortex (M), and normalized analysis of expression levels in each group (N). (*n* = 7 per group). Each data point in the statistical plots represents one biological replicate, and each experiment was performed with three technical replicates. Data were analyzed with one‐way ANOVA followed by Tukey's multiple comparisons test (B, D, E, H, J, L, and N). All data are presented as the mean ± SEM.

To investigate the causes of behavioral abnormalities in the offspring of DSS‐treated dams, we examined the proliferation and differentiation of NSCs in fetal mice. Then, we performed intrauterine electroporation (IUE) assays on embryonic day 13.5 (E13.5) embryonic brains from the vehicle (H_2_O) group, DSS‐treated group, and DSS + *Gsdmd^cko‐Vil1^* group, using GFP plasmids. Compared to the vehicle (H_2_O) and the DSS + *Gsdmd^cko‐Vil1^* group, the DSS‐treated group showed an abnormal distribution of GFP cells (Figure 3A), which was specifically manifested by an increase in the VZ/SVZ and IZ zones, while a decrease in the CP zone (Figure 3B). Additionally, we performed a 24 h BrdU labeling assay and found that compared with the vehicle (H_2_O) group and the DSS + *Gsdmd^cko‐Vil1^* group, the proportion of cell cycle exit (BrdU^+^Ki67^−^) in the fetal brain of the DSS‐treated group was decreased (Figure 3C,D). Furthermore, we also examined the status of the neural stem cell pool. Compared with the vehicle (H_2_O) group and the DSS + *Gsdmd^cko‐Vil1^* group, the DSS‐treated group exhibited an increase in the number of Pax6^+^ cells (Figure S4A,B) and Tbr2^+^ cells (Figure 3E,F). These data indicate that the excessive proliferation of NSCs in the offspring of colitic dams depends on Gsdmd in the maternal colonic epithelium.

Next, we analyzed the status of neurogenesis. We found that on postnatal day 0 (P0), the number of Cux1^+^ (Figure 3G,H) neurons and Satb2^+^ (Figure 3I,J) neurons in the cortex of DSS‐treated offspring was significantly increased, while no significant changes were observed in the number of Ctip2^+^ (Figure S4C,D) neurons and Tbr1^+^ (Figure S4E,F) neurons. Furthermore, on E16.5, immunofluorescence results showed that the expression level of the neuronal marker Tuj1 in the offspring of the DSS‐treated group had already increased at this stage (Figure 3K,L), and the western blot assay also confirmed the increased expression of Tuj1 at E16.5 (Figure S4G,H). In conclusion, we observed that the increase in the number of neurons in the offspring of pregnant mice with colitis could be rescued by knocking out Gsdmd in the colonic epithelium of the pregnant mice.

Additionally, we analyzed the proliferation markers Ki67 and PCNA in the embryonic cerebral cortex, and found that the increased proliferative capacity of the fetal mouse cerebral cortex after DSS treatment depended on Gsdmd in the maternal colonic epithelium (Figure 3M,N).

Additionally, by performing immunofluorescence staining for cleaved‐Caspase3, we detected apoptotic cells in the cortex of offspring from colitis mice and vehicle controls. We found that no difference was observed in the number of apoptotic cells between the two groups (Figure S4I,J). In addition, knockout of Gsdmd in maternal mice under normal drinking water conditions did not affect the generation of fetal brain intermediate progenitor cells (Tbr2) or the expression of the proliferation marker PCNA (Figure S4K,L). Since approximately half of the offspring were heterozygous for Gsdmd knockout following conditional knockout of Gsdmd in maternal mice, we found that heterozygous knockout of Gsdmd in the offspring also did not affect the expression of Tbr2 and PCNA (Figure S4M,N).

In conclusion, we found that maternal colitis leads to increased proliferative capacity of fetal NSCs and the generation of more neurons, and this abnormality could be rescued by the knockout of Gsdmd in the colonic epithelium of maternal mice.

### 2.5 Increased Fetal Microglia are not Associated With Excessive NSC Proliferation

**FIGURE 4:**
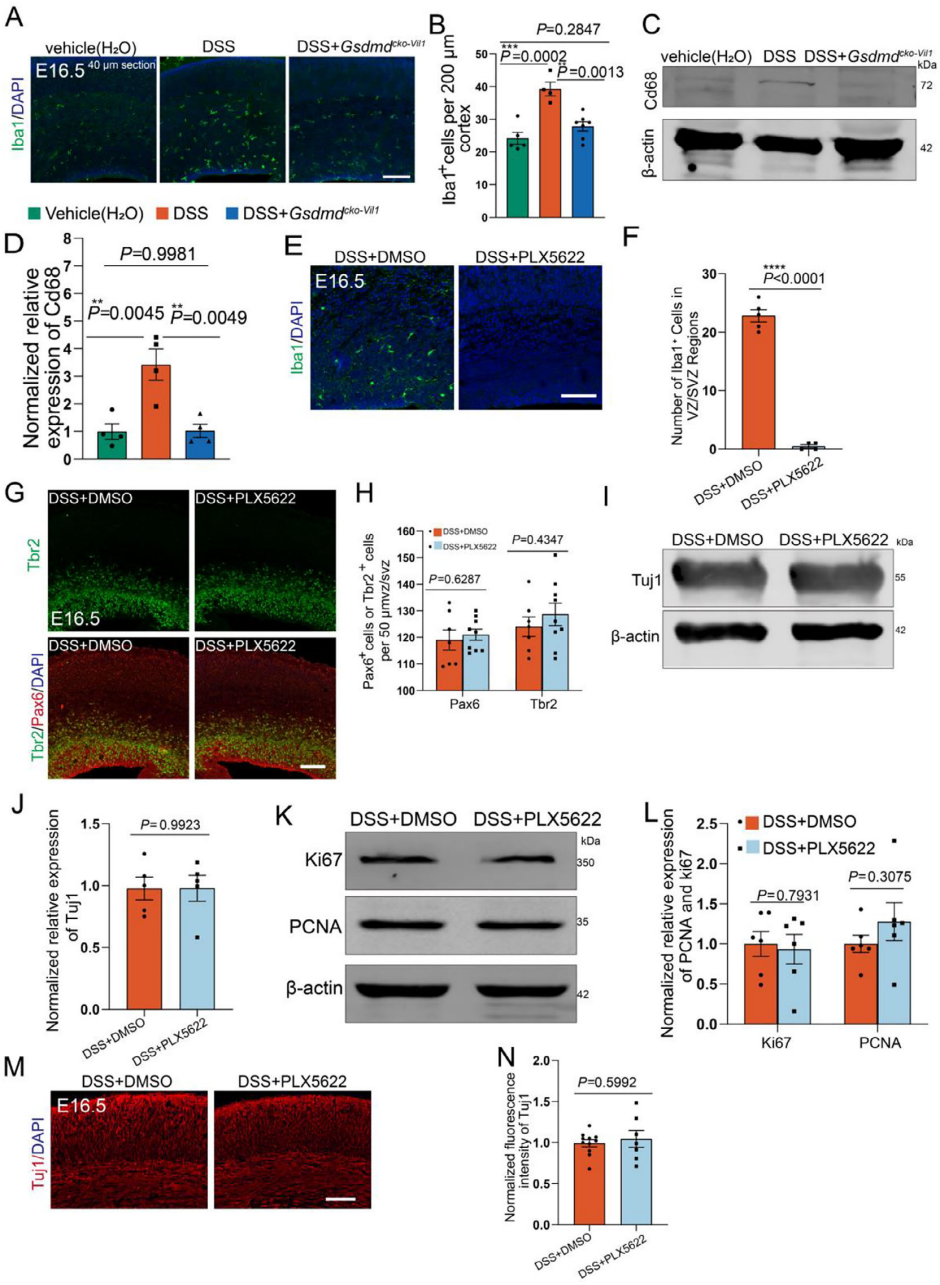
Increased fetal microglia are not associated with excessive NSC proliferation. (A,B) Representative confocal images of microglia at E16.5 (A) and statistical analysis of the number of microglia (B). (*n* = 5, vehicle (H_2_O) group; *n* = 4, DSS group; *n* = 7, DSS + *Gsdmd^cko‐Vil1^* group). Scale bar = 100 µm. (C,D) Representative Cd68 immunoblot images (C) and relative quantitative analysis of Cd68 at E16.5 (D). (*n* = 4 per group). (E,F) Depletion of microglia in the fetal cerebral cortex of maternal mice with colitis using PLX5622 at E16.5 (E), and statistical analysis of the depletion efficiency (F). (*n* = 5, DSS + DMSO group; *n* = 4, DSS + PLX5622 group) Scale bar = 100 µm. (G,H) Representative confocal images of Pax6^+^ and Tbr2^+^ cells at E16.5 following PLX5622 treatment (G), and analysis of their numbers (H). (*n* = 7, DSS + DMSO group; *n* = 9, DSS + PLX5622 group). Scale bar = 100 µm. (I,J) Representative Tuj1 immunoblot images at E16.5 following PLX5622 treatment (I), and quantitative analysis of its relative expression level (J). (*n* = 5 per group). (K,L) Representative PCNA and Ki67 immunoblot images at E16.5 following PLX5622 treatment (K), and quantitative analysis of relative expression levels (L). (*n* = 6 per group). (M,N) Representative confocal images of Tuj1 at E16.5 following PLX5622 treatment (M), and analysis of its relative intensity (N). (*n* = 11, DSS + DMSO group; *n* = 7, DSS + PLX5622 group). Scale bar = 100 µm. Each data point in the statistical plots represents one biological replicate, and each experiment was performed with three technical replicates. Data were analyzed with unpaired two‐tailed *t*‐tests (F, H, J, L, and N), and with one‐way ANOVA followed by Tukey's multiple comparisons test (B and D). All data are presented as the mean ± SEM.

Extracerebral inflammation often perturbs intracerebral immune homeostasis, which is characterized by an increase in the number of microglia. To evaluate the intracerebral immune status in fetuses from colitic dams, we detected the number of microglia in the fetal brain across three groups (the vehicle (H_2_O) group, the DSS‐treated group, and the DSS‐treated + *Gsdmd^cko‐Vil1^* group). We found that the number of microglia was increased in the DSS‐treated group (Figure 4A,B). Additionally, Western blot analysis revealed that the elevated level of Cd68 in the DSS‐treated group was dependent on Gsdmd in the colonic epithelium of maternal mice (Figure 4C,D). In addition, knockout of Gsdmd in pregnant mice under normal drinking water conditions did not result in differences in the number of fetal cortical microglia (Figure S5A,B). Furthermore, heterozygous knockout of Gsdmd in the colonic epithelium of fetal mice also did not lead to differences in the number of microglia (Figure S5C,D).

To investigate whether the excessive proliferation of NSCs is caused by the abnormal increase of microglia in the brains of fetal mice, we treated maternal mice with PLX5622 from embryonic day 9.5 (E9.5) to E16.5 to deplete microglia in the embryonic brain. Furthermore, the measurement of Iba1‐positive cell numbers in the fetal mouse brains confirmed a significant depletion of microglia (Figure 4E,F). We found that, in the context of maternal colitis, depletion of microglia had no effect on the number of Pax6‐positive and Tbr2‐positive cells, the major neural stem/progenitor cell population (Figure 4G,H). Additionally, Western blot results showed that at E16.5, the level of Tuj1 remained unchanged (Figure 4I,J), and the expression levels of Ki67 and PCNA were also unchanged (Figure 4K,L). Furthermore, immunofluorescence analysis at embryonic day 16.5 (E16.5) also confirmed that the depletion of microglia failed to rescue the overexpression of Tuj1 in the fetuses of maternal mice with colitis (Figure 4M,N).

Given that the target of PLX5622 is widely distributed, to consolidate the above results, we also constructed mice with microglia‐specific expression of diphtheria toxin receptor (DTR) (Figure S5E), and achieved more specific depletion of microglia via regular intracerebroventricular injection of diphtheria toxin (DT) (Figure S5F). After diphtheria toxin injection and immunofluorescence staining for Iba1, we found that this method could also significantly deplete microglia in the embryonic brain (Figure S5G,H). Subsequently, by detecting the expression levels of Pax6, Tuj1, Ki67, and PCNA in the brains of offspring from colitis mice by Western blot analysis (Figure S5I), we found that the depletion of microglia did not reduce the expression of these proteins (Figure S5J).

Taken together, we found that maternal colitis disrupts immune homeostasis in the fetal brain, and this disruption could be rescued by the knockout of Gsdmd in the colonic epithelium of maternal mice. However, the previously described excessive proliferation of NSCs was not caused by the increase in microglia.

### 2.6 LPS Translocation Caused by Intestinal Barrier Leakage in Maternal Mice Leads to the Activation of Cortical Microglia in Fetal Mice

**FIGURE 5:**
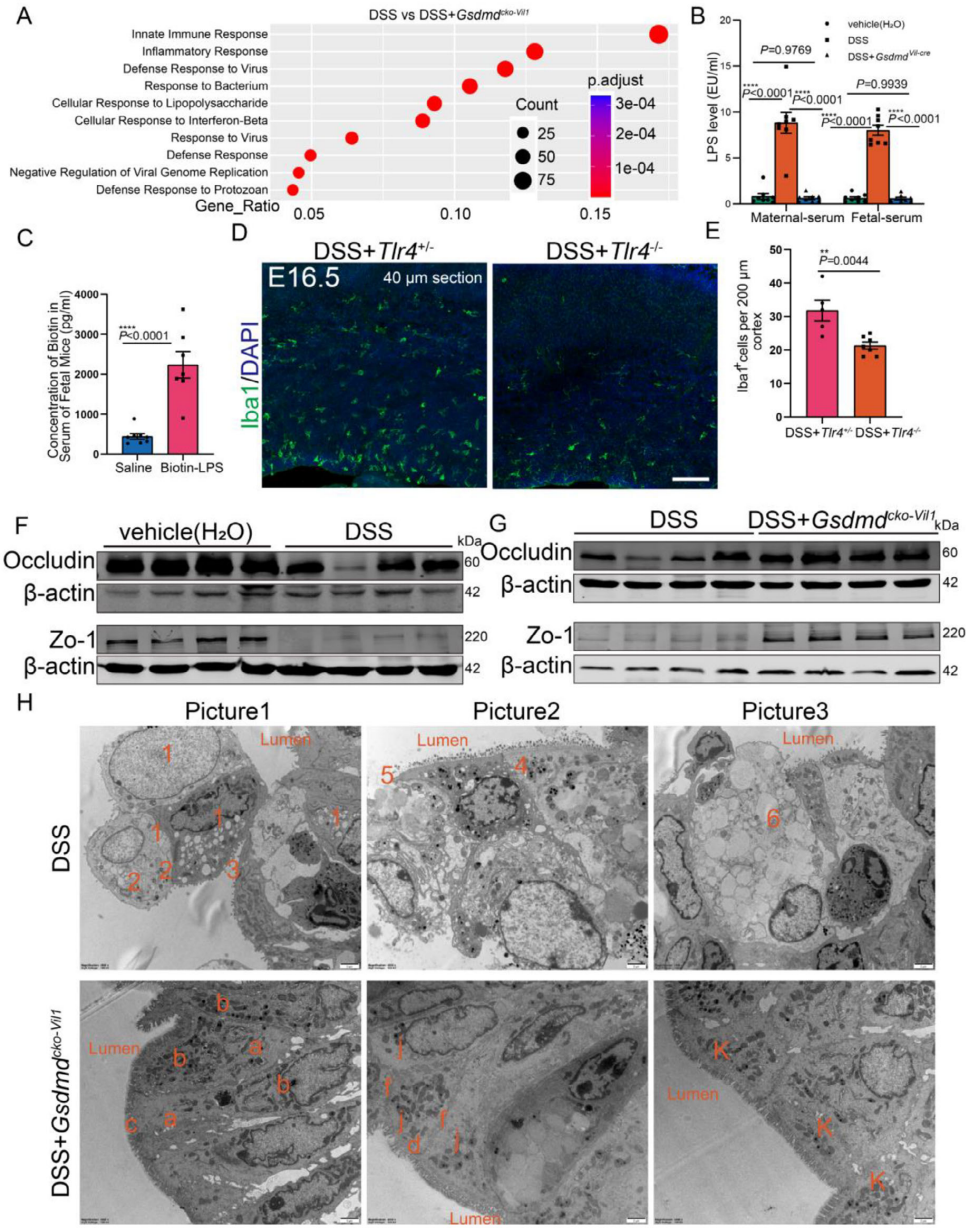
LPS translocation caused by intestinal barrier leakage in maternal mice leads to the activation of cortical microglia in fetal mice. (A) The GO Biological Process analysis revealed the top ten enriched pathways. (*n* = 3 per group). (B) The determination of LPS levels in the serum of pregnant mice and fetal mice using the LAL assay. (*n* = 8 per group). (C) Determination of biotin content in fetal mouse serum using the ELISA method. (*n* = 8, saline group; *n* = 7, biotin‐LPS group). (D,E) Representative immunofluorescence images of microglia (D), and statistical analysis of microglial numbers (E). (*n* = 5, DSS + *Tlr4* ^+/−^; *n* = 7, DSS + *Tlr4* ^−/−^). Scale bar = 100 µm. (F,G) Western blot images of colonic Occludin and Zo‐1. (*n* = 4 per group). (H) Colonic electron microscopy images of the DSS group and DSS + *Gsdmd^cko‐Vil1^* group. (1, pyroptotic epithelial cell; 2, mitochondrial damage; 3, reduction of microvilli; 4, disappearance of junctions; 5, cell lysis; 6, cell swelling. a, epithelial cell; b, mitochondria; c, microvilli; d, tight junction; f, adherent junction; j, intact cell; k, normal cell volume.). Each data point in the statistical plots represents one biological replicate. For experiments (B, C, D, E, F, G, and H) the number of technical replicates was 3. For experiment A, the number of technical replicates was 1. Data were analyzed with unpaired two‐tailed *t*‐tests (C and E), and with one‐way ANOVA followed by Tukey's multiple comparisons test (B). All data are presented as the mean ± SEM.

To investigate how Gsdmd knockout in the intestinal epithelium of maternal mice rescues colitis‐induced excessive proliferation of NSCs and cortical immune imbalance in offspring, we performed transcriptome sequencing analysis on the cortex of offspring from DSS‐treated wild‐type C57/BL6 and *Gsdmd^cko‐Vil1^* pregnant mice. Gene Ontology (GO) analysis revealed that the differentially expressed genes were enriched in LPS response‐related and immune response‐related processes (Figure 5A). We confirmed by qPCR that the canonical downstream response genes of LPS, IL‐1β, and IL‐6 were downregulated following Gsdmd knockout in maternal mice (Figure S6A). Then, we measured the LPS levels in the plasma of maternal mice and fetal mice using the Limulus amebocyte lysate (LAL) assay, and found that compared with the vehicle (H_2_O) group and the DSS + *Gsdmd^cko‐Vil1^* group, the LPS levels in the DSS‐treated group showed a significant increase in the plasma of both maternal mice and fetal mice (Figure 5B). We analyzed the placental penetration of LPS via the injection of biotin‐labeled LPS and found that following the injection of biotin‐labeled LPS into pregnant mice with colitis, the biotin level in fetal plasma increased significantly (Figure 5C). Compared with *Tlr4* (a key mediator of LPS) heterozygous knockout fetal mice, we found that *Tlr4* knockout mice resulted in a decreased number of microglia in fetal mice under maternal colitis conditions (Figure 5D,E). Meanwhile, loss of *Tlr4* in fetal mice under normal drinking water conditions did not result in differences in the number of microglia (Figure S6B,C). This suggests that the increase in microglia is caused by LPS translocation; however, by Western blot analysis of markers (PCNA, Tuj1, Tbr2, Pax6) for the proliferation and differentiation of NSCs, we observed that *Tlr4* knockout failed to rescue the excessive proliferation of fetal NSCs induced by maternal colitis (Figure S6D,E). We also examined the neuronal markers Tuj1 and NeuN in the cortex of postnatal day 0 (P0) mice and found that *Tlr4* knockout did not prevent the excessive production of neurons (Figure S6F–H). Subsequently, through immunostaining of the cortex, we found that *Tlr4* was mainly localized to microglia (Figure S6I). Overall, LPS induces an increase in fetal microglia rather than the excessive proliferation of neural stem cells.

To investigate why knocking out Gsdmd in the colonic epithelium of pregnant mice rescued the elevation of LPS levels in colitic pregnant mice and their fetuses, we performed Western blot experiments on the junction proteins of the maternal colonic epithelium. We found that compared with the vehicle (H_2_O) group, the expression levels of Occludin and Zo‐1 were decreased in the DSS‐treated group, and this decrease could be rescued by knocking out Gsdmd in the colonic epithelium (Figure 5F,G). In addition, conditional knockout of colonic epithelial Gsdmd in pregnant mice did not result in differences in the expression of the colonic epithelial tight junction marker Occludin (Figure S6J,K). To further confirm these results, we performed transmission electron microscopy (TEM) imaging on the colons of mice in the DSS‐treated group and the DSS + *Gsdmd^cko‐Vil1^* group. Compared with the DSS‐treated group, the DSS + *Gsdmd^cko‐Vil1^* group exhibited an increased number of colonic apical villi, reduced apical cell death, and enhanced strength of intercellular junctions (Figure 5H).

Overall, Gsdmd in the colonic epithelium during colitis mediates the translocation of LPS from the intestinal lumen to the fetal brain, leading to an increase in microglia; however, it is not the cause of enhanced proliferation of NSCs.

### 2.7 Maternal Gsdmd‐Mediated Epithelial IL‐33 Release During Colitis Promotes Neural Proliferation via the St2 Receptor Within the Fetal Mouse Brain

**FIGURE 6:**
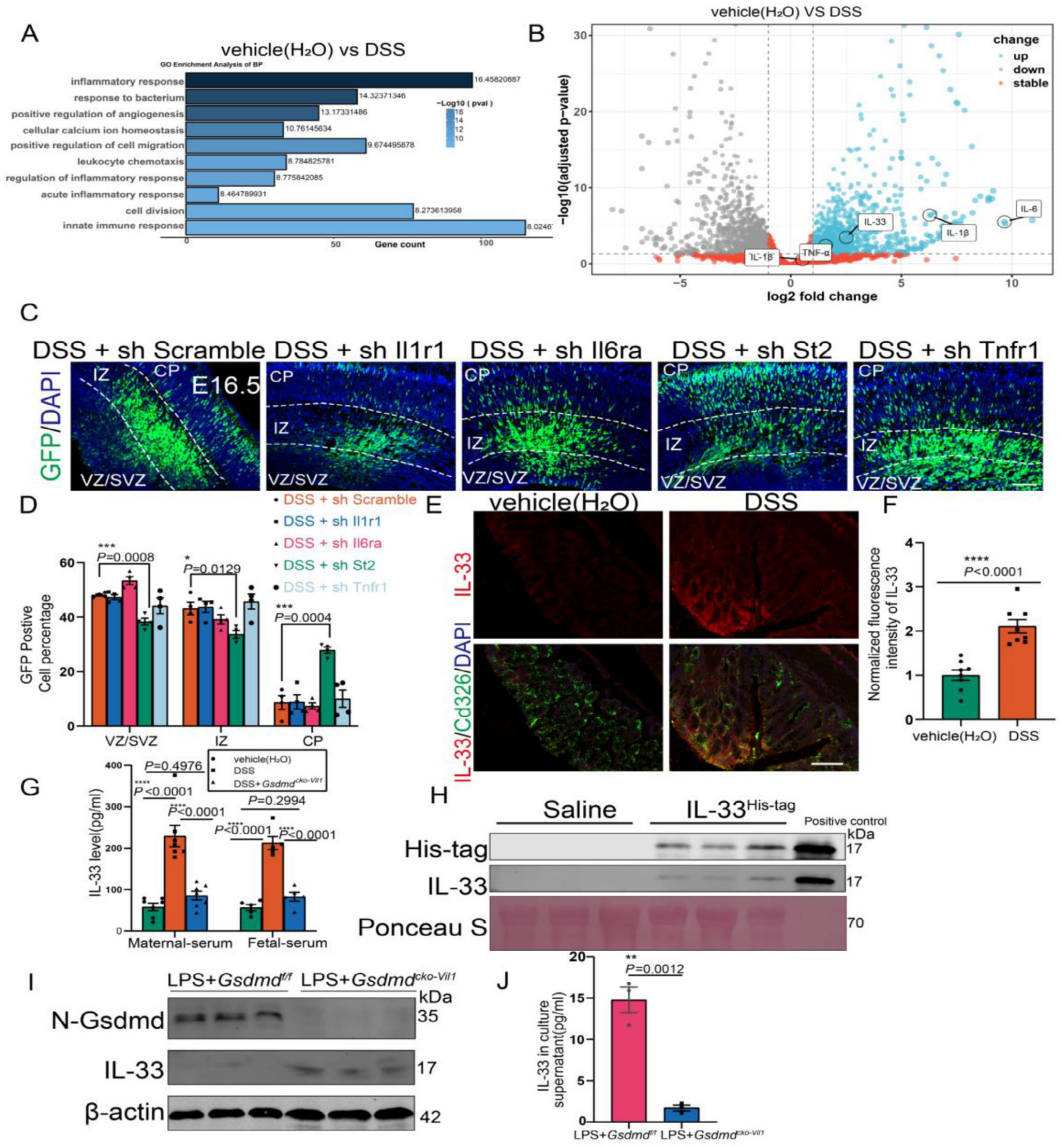
Maternal Gsdmd‐mediated epithelial IL‐33 release during colitis promotes neural proliferation via the St2 receptor within the fetal mouse brain. (A) The top 10 GO terms enriched by differentially expressed genes via GO enrichment analysis. (B) The volcano plot shows the differentially expressed genes. (C,D) Representative images of GFP distribution (C), and the percentage distribution of GFP in the VZ/SVZ, IZ, and CP regions (D). (*n* = 4 per group). (sh Scramble vs sh St2: CP regions: R^2^ = 0.89, 95% confidence interval of the difference: 12.50 to 25.98; IZ regions: R^2^ = 0.67, 95% confidence interval of the difference: ‐16.10 to ‐2.837; VZ/SVZ regions: R^2^ = 0.87, 95% confidence interval of the difference: ‐15.12 to ‐6.576). Scale bar = 100 µm. (E,F) Representative IL‐33 immunofluorescence images (E) and analysis of IL‐33 expression intensity (F). (*n* = 8 per group). Scale bar = 100 µm. (G) ELISA assay for detecting the levels of IL‐33 in the serum of pregnant mice and fetuses. (*n* = 7 for each pregnant group; *n* = 5 for each fetal group). (H) Western blotting images show the presence of His‐tag and IL‐33 in fetal mouse serum. (*n* = 3 per group). (I,J) Western blotting images show the levels of IL‐33 in cell lysates from the WT group and the Gsdmd‐knockout group (I), while the ELISA was used to detect the levels of IL‐33 in the culture supernatant (J). (*n* = 3 per group). Each data point in the statistical plots represents one biological replicate. For experiments (C, D, E, F, G, H, I, and J), the number of technical replicates was 3. For experiments A and B, the number of technical replicates was 1. Data were analyzed with unpaired two‐tailed *t*‐tests (D, F, and J), and with one‐way ANOVA followed by Tukey's multiple comparisons test (G). All data are presented as the mean ± SEM.

To investigate how inflammatory factors produced by colitis affect the fetal brain, we performed RNA‐seq on colonic epithelial cells from DSS or vehicle‐treated maternal mice. We found that the biological processes identified by Gene Ontology analysis were mainly enriched for inflammatory response and bacterium‐responsive pathways. (Figure 6A). Since we have already demonstrated that LPS and the subsequent increase in microglia were not the causes of excessive proliferation of NSCs, we focused on the cytokines produced by epithelial cells during colitis. We identified four cytokines: TNF, IL‐33, IL‐6, and IL‐1β (Figure 6B). Additionally, we verified by qPCR that the expression of these four pyroptosis‐related cytokines was indeed upregulated (Figure S7A). Next, we constructed receptor knockdown plasmids capable of knocking down the receptors corresponding to these four cytokines (Figure S7B). Subsequently, we conducted in utero embryonic electroporation experiments on the E13.5 fetal brains of pregnant mice with colitis using four types of knockdown plasmids and found that knocking down St2 (the receptor for IL‐33) significantly improved the distribution of GFP‐positive cells (Figures 3A,B and 6C,D). Combining immunofluorescence and Western blotting, we found that St2 was mainly expressed in the VZ/SVZ regions (Figure S7C), and its expression level gradually decreases as embryonic development progresses (Figure S7D). We also used co‐immunoprecipitation (Co‐IP) to verify the interaction between IL‐33 and St2 (Figure S7E). Knocking down St2 using the intrauterine IUE technique resulted in cell cycle arrest (Figure S7F,G). This suggests that St2 also plays a regulatory role in normal brain development.

Then, we performed immunofluorescence staining targeting IL‐33 on the colons of DSS or vehicle‐treated pregnant mice, and we found that the expression of IL‐33 in the colonic epithelium was significantly increased after DSS treatment (Figure 6E,F). Subsequently, we used the ELISA to detect IL‐33 in the serum of pregnant mice and fetal mice. We found that compared with the vehicle (H_2_O) group, DSS treatment led to an increase in serum IL‐33 levels in both pregnant mice and fetal mice, while knocking out Gsdmd in the colonic epithelium of pregnant mice reduced the serum IL‐33 levels (Figure 6G). Next, to evaluate the placental permeability of IL‐33, we injected His‐tagged IL‐33 into the tail veins of pregnant mice with colitis. Following this, Western blotting analysis revealed that the levels of both His‐tag and IL‐33 in fetal mouse serum were increased (Figure 6H). Gsdmd can mediate the release of IL‐1β from colonic epithelial cells [15], but the relationship between Gsdmd and IL‐33 remains unclear. LPS, as a direct inducer of pyroptosis, can stimulate cytokine release from cells cultured in vitro [16]. We cultured wild‐type and Gsdmd‐knockout colonic epithelial cells in vitro and treated them with LPS stimulation. We found that knocking out Gsdmd increased the level of IL‐33 in the cell lysates (Figure 6I), while it decreased the level of IL‐33 in the culture supernatant (Figure 6J). Similarly, disulfiram, a pore‐forming inhibitor of Gsdmd, could also block the release of IL‐33 (Figure S7H,I). This suggests that IL‐33 is released via Gsdmd.

Taken together, we found that the expression level of IL‐33 in the colonic epithelium increased during colitis, and IL‐33 is released extracellularly through Gsdmd pores. Furthermore, knocking down St2 can rescue the abnormal distribution of GFP‐positive cells in the fetal mouse brain.

### 2.8 IL‐33 Increases NSCs and Induces Autistic‐Like and Anxiety‐Like Behaviors

**FIGURE 7:**
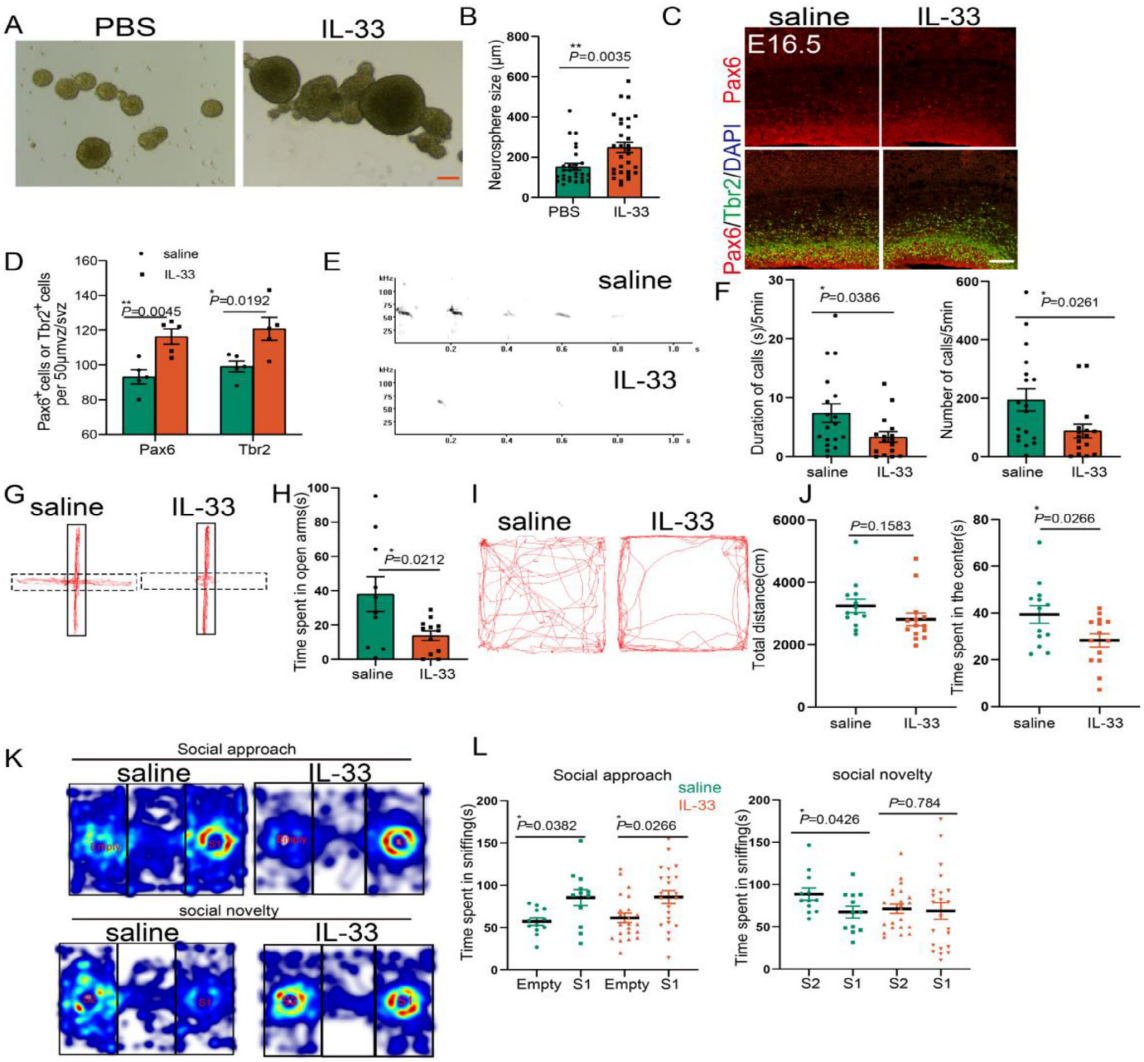
IL‐33 increases NSCs and induces autistic‐like and anxiety‐like behaviors. (A,B) Representative images of neurospheres after stimulation with IL‐33 or vehicle (A) and the statistical analysis of neurosphere diameters in the two groups (B). (For the PBS group, biological replicates: *n* = 28 from six independent experiments; for the IL‐33 group, biological replicates: *n* = 30 from six independent experiments). Scale bar = 100 µm. (C,D) Representative Pax6 and Tbr2 immunofluorescence images in fetal mouse cortex after IL‐33 or vehicle injection (C), and the statistical graph quantifying the numbers of positive cells (D). (Biological replicates: *n* = 5 per group, technical replicates = 3). Scale bar = 100 µm. (E,F) Representative ultrasonic vocalization spectrograms of pups at P5 (E), along with statistical graphs of the number and duration of ultrasonic vocalizations (F). (*n* = 18, saline group; *n* = 16, IL‐33 group). (G,H) Representative trajectory maps of mice exploring the elevated plus maze (G), and time spent in the open arms (H). (*n* = 10, saline group; *n* = 12, IL‐33 group). (I,J) Representative trajectory maps of mice in the open field test (I), and time spent in the central zone (J). (*n* = 13, saline group; *n* = 14, IL‐33 group). (K,L) Representative trajectory heatmaps of mice (K), and the duration of mice's exploration in each cage (L). (*n* = 12, saline group; *n* = 22, IL‐33 group). Each data point in the statistical plots represents one biological replicate. The number of technical replicates for the behavioral experiments was 1. Data were analyzed with unpaired two‐tailed *t*‐tests (B, D, F, H, J, and L). All data are presented as the mean ± SEM.

To further clarify the impact of IL‐33 on brain development, we cultured NSCs in vitro and found that after adding IL‐33, the NSCs formed larger neurospheres (Figure 7A,B). We injected IL‐33 into the fetal brains at E13.5 and analyzed the number of Pax6^+^ and Tbr2^+^ cells at E16.5. We found that IL‐33 led to an increase in the number of these two types of cells (Figure 7C,D). During in vitro culture of neural stem cells, the addition of IL‐33 significantly increased the proportion of PCNA‐positive neural stem cells. This suggests that IL‐33 can promote the proliferation of neural stem cells (Figure S8A,B). In addition, we used an IL‑33 neutralizing antibody to reduce the level of IL‑33 in fetal serum (Figure S8C). After neutralization of IL‐33, the excessive expansion of the neural stem cell pool induced by colitis in maternal mice was significantly attenuated, and the expression of the proliferation marker PCNA was also decreased (Figure S8D,E). Meanwhile, the expression level of the neuronal marker Tuj1 in the offspring of maternal mice with colitis was decreased after neutralization of IL‐33 (Figure S8F,G).

Subsequently, we conducted behavioral tests on the offspring of mice injected with IL‐33. We found that mice treated with IL‐33 at the embryonic stage exhibited fewer counts and shorter durations of ultrasonic vocalizations (Figure 7E,F). This suggests that IL‐33‐treated fetal mice exhibit impairments in communication with the external environment after birth. In the elevated plus maze test, mice treated with IL‐33 showed decreased exploration time in the open arms (Figure 7G,H); in the open field test, they also exhibited decreased exploration time in the central zone (Figure 7I,J). These suggest that the IL‐33‐treated offspring exhibit anxiety‐like behaviors. Finally, we evaluated the changes in social behavior of mice after IL‐33 treatment. After the test mice had acclimated for 10 min, a same‐sex stranger mouse (Stranger 1) was placed into one side of the cage. We then found that both the IL‐33‐treated group and the saline group preferred to approach cages containing a stranger mouse rather than empty cages. After introducing another stranger mouse (Stranger 2), mice in the saline group spent more time exploring Stranger 2, whereas the IL‐33‐treated mice spent almost the same amount of time exploring Strangers 1 and 2 (Figure 7K,L), which indicates that IL‐33 treatment leads to impairments in the mice's social recognition memory. Furthermore, these behavioral deficits are highly similar to those observed in the offspring of pregnant mice with colitis.

Taken together, IL‐33 leads to an expansion of the neural stem cell population and induces autistic‐like and anxiety‐like behaviors. These behavioral abnormalities, along with the phenotype of abnormal neural stem cell proliferation, are highly similar to those observed in the offspring of pregnant mice with colitis.

## 3 Discussion

In this study, we investigated how colitis in pregnant mice leads to autistic‐like behaviors in offspring. We confirmed that IL‐33 released from maternal colonic epithelial cells through Gsdmd pores during experimental colitis drives cortical developmental defects in fetal mice. Either knocking out Gsdmd in the colonic epithelium of pregnant mice or knocking down St2 in the fetal brain can rescue the abnormal distribution of GFP‐positive cells induced by DSS treatment. These results demonstrate that the Gsdmd‐IL‐33‐St2 axis is a central pathway linking maternal colitis to fetal brain injury.

Epithelial‐specific Gsdmd deletion restored the number of colonic apical epithelial cells, but it did not normalize the overall colitis pathology score. Whether knockout of Gsdmd can alleviate the pathological score of colitis remains inconclusive [15, 17, 18, 19]. We also detected substantial expression of Gsdma, Gsdmc, and Gsdme in colonic epithelial cells. This suggests that different Gasdermins may contribute to colitis at distinct stages. Notably, epithelial Gsdmd deletion partially improved colitis pathology after 4 days of 2.5% DSS treatment.

We found that the offspring of pregnant mice with colitis exhibit abnormal brain development and behavioral deficits. Such a phenomenon is not uncommon; for example, studies have shown that maternal Poly(I:C) exposure also impairs embryonic brain development [3, 4, 20]. Stimulating pregnant mice with *Toxoplasma gondii* antigen leads to behavioral abnormalities in their offspring [6, 21, 22]. Together, these studies suggest that diverse maternal inflammatory challenges during pregnancy can perturb fetal neurodevelopment.

We found evidence of intestinal barrier disruption in colitis, accompanied by elevated LPS in the maternal and fetal circulation. This was associated with an increased number of fetal microglia. Previous studies have confirmed that LPS injection during pregnancy induces an increase in the number of microglia [23] and a decrease in the body weight of newborn mice [24, 25]. These are consistent with our findings.

Gsdmd pore formation facilitates the release of proinflammatory cytokines during pyroptosis, including IL‐1β, IL‐18, and IL‐33 [26, 27, 28, 29]. Knockout or inhibition of Gsdmd can improve the prognosis and survival rate of mice in an inflammatory state. The activation of Gsdmd promotes the development of respiratory inflammation caused by COVID‐19 and influenza A [30, 31, 32, 33]. Knocking out Gsdmd or inhibiting Gsdmd pore formation can significantly improve the prognosis and survival rate of mice with sepsis [13, 34, 35, 36, 37]. Consistent with this, epithelial Gsdmd deletion markedly reduced IL‐33 release and rescued abnormal fetal brain development and offspring behavioral deficits.

We found that the expression of IL‐33 is upregulated in colonic epithelial cells during colitis, while St2—the receptor for IL‐33—is mainly present in the VZ/SVZ of the embryo. The excessive presence of IL‐33 causes the expansion of the neural stem cell pool and leads to postnatal behavioral deficits. In a neonatal hypoxia–ischemia model, supplementation with IL‐33 promotes neural stem cell survival and proliferation, thereby expanding the neural stem cell pool [38]. Previous studies have reported that the binding of IL‐33 to St2 can regulate the synaptic phagocytic function of microglia [39, 40, 41]. In this study, we focused on NSC proliferation. This choice was motivated by the enrichment of St2 in the embryonic VZ/SVZ. In contrast, microglia‐mediated synaptic pruning occurs primarily after birth [42, 43].

Taken together, our findings show how maternal colitis perturbs fetal neurodevelopment and identify epithelial Gsdmd‐dependent IL‐33 release as a key driver of these defects.

## 4 Conclusion

In conclusion, our study demonstrates that maternal colitis during pregnancy in mice enhances fetal neural stem cell proliferation and increases microglial numbers in the developing brain, ultimately leading to social deficits and anxiety‐like behaviors in the offspring. These abnormalities are driven by elevated levels of IL‐33 in the maternal intestine, a process mediated by epithelial Gsdmd. Our findings identify maternal intestinal epithelial Gsdmd and its downstream immune mediators as key regulators linking colitis to neurobehavioral disturbances in the offspring.

## 5 Materials and Methods

### 5.1 Animals

All animal experiments were approved by the Animal Care and Use Committees of the Institute of Zoology, Chinese Academy of Sciences (Ethical License Number: #IOZ2023154). *Vil1‐Cre* mice (B6.Cg‐Tg(*Vil1‐cre*)1000Gum/J, JAX stock: #021504) and *Cx3cr1‐Cre* mice (B6J.B6N(Cg)‐*Cx3cr1*tm1.1(cre)Jung/J, JAX stock: #025524) were purchased from Jackson Laboratory. *Rosa26‐iDTR* mice were purchased from Cyagen. *Gsdmd^fl/fl^* mice were purchased from RIKEN BRC. *Tlr4*‐KO mice were generously donated by Professor Zou Haidong, Shanghai First People's Hospital. C57BL/6 mice were purchased from Charles River.

### 5.2 DSS‐Induced Colitis

Pregnant mice at E9.5 were randomly assigned to receive either 2.5% DSS‐containing drinking water or normal drinking water. After 4 consecutive days of this treatment, the drinking water was replaced with normal drinking water.

### 5.3 Propidium Iodide (PI) Staining

After anesthetizing the mice to be tested, we injected Propidium Iodide (PI) dye at a concentration of 100 µg/mL into the colons of the mice. Thirty seconds later, the mice were euthanized. The colons were rinsed thoroughly with physiological saline and immersed in 4% paraformaldehyde for 1 min, followed by immediate frozen sectioning.

### 5.4 Isolation of Colonic Epithelial Cells

After euthanizing the mice, the colons were longitudinally incised and rinsed with PBS. The colonic tissues were cut into approximately 1 mm × 1 mm × 1 mm pieces, which were then incubated in Gentle Cell Dissociation Reagent (STEMCELL TECHNOLOGIES, Cat# 100–0485) at 37°C for 20 min. Subsequently, the samples were washed with 2 mm EDTA in PBS, and the supernatant was collected. After filtering through a 70 µm strainer, the cells were labeled with PE‐conjugated anti‐Cd45 and FITC‐conjugated anti‐Cd326 antibodies at 4°C for 30 min in the dark. Colonic epithelial cells (Cd326^+^, Cd45^−^) were sorted by flow cytometry.

### 5.5 Microglia Ablation Experiment

For microglial ablation induced by PLX5622, pregnant mice were treated at a dose of 50 mg/kg starting on embryonic day (E) 10.5 for a total of 6 consecutive days. For microglial depletion using diphtheria toxin (DT), DT (5 ng/nL) was injected into the fetal lateral ventricle starting on E13.5 for 3 days at a volume of approximately 1 µL.

### 5.6 In Utero Electroporation

Pregnant mice at E13.5 were anaesthetized by the intraperitoneal injection of 2,2,2‐tribromoethanol (Sigma–Aldrich, Cat# T48402), and the uterine horns were exposed. About 1 µL plasmid DNA (1500 ng/µl) mixed with Fast Green (Sigma–Aldrich, Cat# F7258) was gently injected into the embryonic lateral ventricles with a heat‐pulled glass micropipette. Embryos were subjected to five 50 ms pulses (950 ms interval time between each pulse) at 35 V generated by an electroporator (BTX ECM830). Three days later, the brains of fetal mice were taken out for analysis of the distribution ratio of neural cells.

### 5.7 Cell Cycle Exit Detection

BrdU was dissolved in normal saline at a final concentration of 10 mg/mL. Pregnant mice at 15.5 days of gestation were intraperitoneally injected with BrdU at a dose of 50 mg/kg. The mice were sacrificed 24 h later, and the brains of the fetal mice were collected for immunofluorescence staining of BrdU and Ki67. The proportion of BrdU‐single‐positive cells among BrdU‐positive cells was counted to obtain the cell cycle exit rate.

### 5.8 Immunostaining

Fix 15 µm or 40 µm sections in 4% PFA for 30 min, then block with 5% BSA, dilute the antibody (see Table S4 for details) with 1% BSA, and incubate overnight at 4°C. After removing the primary antibody, add the secondary antibody (see Table S4 for details) diluted with 1% BSA and incubate at room‐temperature for 1 h. Finally, use DAPI (2 µg/mL, Sigma‐Aldrich, Cat# D9542) to stain the cell nucleus. During the above process, when changing the liquid on the glass slide, except before adding the primary antibody, wash three times with 1% PBST (1% Triton X‐100 in PBS) for 10 min each time.

### 5.9 Western Blotting

The tissue or cells were lysed by RIPA containing PMSF and protease inhibitor cocktail. Protein concentration was measured by the BCA protein concentration assay. The protein concentration was measured using the BCA protein assay method. Subsequently, these proteins were separated by 12% or 10% SDS‐PAGE and transferred onto nitrocellulose membranes (NC), then incubated with primary antibodies (see Table S4 for details) overnight. Next day, the membranes were incubated with secondary antibodies (see Table S4 for details) for 1 h at room‐temperature.

### 5.10 Behavioral Tests

Mouse pups P5 were used for Ultrasonic vocalization, and 8‐10‐week‐old adult mice were used for other behavioral experiments. Avisoft RECORDER 4.4.1‐24, Avisoft SASLab Pro 5.3.2‐20, and Noldus EthoVison XT 14.0 were used for data collection.

### 5.11 Mouse Ultrasonic Vocalization Test

In a quiet, warm room, 5‐day‐old mouse pups were placed in a sound‐attenuating chamber. The ultrasonic recording probe was positioned above the pups. Ultrasonic vocalizations were recorded using Avisoft RECORDER 4.4.1‐24 and analyzed using Avisoft SASLab Pro 5.3.2‐20. The number and duration of pup ultrasonic vocalizations were measured.

### 5.12 Open Field Test

The mice were placed in a box (100 cm × 100 cm × 100 cm). Locomotor trajectory data of mice were recorded for 5 min.

### 5.13 Self‐Grooming

Mice were individually placed into a novel testing cage equipped with fresh bedding material. After a 5 min habituation period, behavioral monitoring was conducted over the subsequent 10 min interval using a video camera. Manual quantification of the cumulative duration for facial, cranial, or generalized body scratching/rubbing behaviors was performed via a digital stopwatch.

### 5.14 Three‐Chamber Social Assay

The test apparatus comprised three equal chambers (20 cm × 45 cm × 30 cm). The tested mice could enter each chamber through the doors. First, the mice were placed in the middle chamber of the box and allowed to explore freely for 10 min to get habituation. Next, a conspecific (C57BL/6) and age‐matched stranger mouse (stranger1) of the same sex was confined in one side of the chambers, while the other chamber remained unoccupied. The duration during which the tested mouse visited the strange mouse within a 10 min exploration period was recorded. Finally, a mouse (Stranger2) of the same sex and age was added to the other empty chamber, and record the duration the tested mouse spent exploring both mice in 10 min.

### 5.15 Elevated Plus Maze Test

The maze consists of two open arms (35 cm × 5 cm) and two enclosed arms of identical size, each equipped with 15‐cm‐high walls. All arms and the central platform are constructed from blue plastic plates and elevated to a height of 60 cm above the ground. During testing, mice were placed on the central platform facing an open arm and allowed to freely explore the maze for 5 min. The duration that a mouse spent in the open arms was monitored and analyzed using Ethovision XT software.

### 5.16 Forced Swim Test

On the first day, mice were transferred to an experimental water tank and permitted to undergo a 10 min free‐swimming session to reduce stress responses. On the subsequent day, animals were re‐exposed to the same swimming protocol for a 10 min duration, during which behavioral parameters, including swimming and climbing actions, were systematically observed and documented in the last 5 min. This experiment was scheduled to be conducted last to avoid affecting the mice.

### 5.17 Electron Microscopy

The colons were fixed using a solution of 2.5% (vol/vol) glutaraldehyde in phosphate buffer (PB) (0.1 m, pH 7.4). This was followed by two washes in PB and two additional washes in deionized water. Subsequently, the colons were immersed in a mixture consisting of 1% (wt/vol) OsO4 and 1.5% (wt/vol) potassium ferrocyanide in an aqueous solution at 4°C for 2 h. After washing, the colons underwent dehydration through a graded alcohol series (30, 50, 70, 80, 90, 100%, and 100%, 10 min each), followed by two 10 min immersions in pure acetone. The samples were then infiltrated in a graded mixture (3:1, 1:1, 1:3) of acetone and SPI‐PON812 resin (21 mL SPI‐PON812, 13 mL DDSA, and 12 mL NMA). This was followed by incubation in pure resin. Finally, the colons were embedded in pure resin containing 1.5% BDMA and polymerized for 12 h at 37°C, followed by an additional 48 h at 60°C. Ultrathin sections (70 nm thick) were obtained using a microtome (Leica EM UC6), These sections were double‐stained with uranyl acetate and lead citrate and examined using a transmission electron microscope (FEI Tecnai Spirit120kV) equipped with an EMSIS CCD camera (VELETA).

### 5.18 Mouse Serum Preparation

Mice were anaesthetized by the intraperitoneal injection of 2,2,2‐tribromoethanol (Sigma–Aldrich, Cat# T48402). The blood from mice was collected by cardiac puncture into a sterile, pyrogen‐free centrifuge tube. After standing at room‐temperature for 1 h, centrifuge it at 3000 rpm at 4°C for 5 min, and then collect the supernatant.

For the collection of fetal mouse serum: After the pregnant mouse was euthanized by carbon dioxide, the fetuses were quickly removed from the pregnant mouse, and the excess fluid on their bodies was blotted dry. Then, a small incision was made in the liver of each fetus, and the fetal body was gently squeezed to ensure sufficient blood flow. The blood was collected using a capillary tube.

### 5.19 Neurosphere Formation Assay

The cerebral cortex was isolated from fetal brains at embryonic day 13.5. These tissues were then digested into single cells using papain. The single cells were seeded into culture dishes at a density of 40 cells/mL of medium and cultured under suspension conditions. On the 3rd day of culture, either IL‐33 (5 ng/mL) or PBS was added to the cultures, and the neurosphere size was observed 4 days later.

### 5.20 Bulk RNA Sequencing

Total RNA was extracted from fetal mouse cortex or from colonic epithelial cells of pregnant mice using TRIzol. RNA quality was assessed using an Agilent 2100 Bioanalyzer. High‐throughput sequencing was performed on an Illumina HiSeq 6000 platform (Annorad Genomics). Quality filtering was carried out using FastQC, and read alignment was performed using Hisat2. Genes with a fold change > 2 and a *P* value < 0.05 were considered differentially expressed.

The released data are available in NCBI GEO under accession numbers GSE310969 and GSE310970. The public sequencing data used in the study were obtained from the NCBI GEO database under the accession number GSE168053.

### 5.21 Single‐Cell Data Analysis

The normalized expression matrix used in the analysis was obtained from the NCBI GEO database, with the accession number GSE116222. The marker genes used for cell clustering are provided in Table S3. Cell types were annotated using a marker‐based rule approach: for each cell, the mean expression of predefined marker genes for each cell type was calculated, and the cell was assigned to the cell type with the highest mean marker expression. GSDMD expression levels were extracted from the annotated data, and differential significance was assessed using the Wilcoxon rank‐sum test.

### 5.22 In Vitro Culture of Colonic Epithelial Cells

Colonic epithelial cells sorted by flow cytometry were seeded into pre‐coated culture plates. After the cells had attached to the plate, they were cultured in medium containing 1 µg/mL LPS for 12 h. Subsequently, the levels of IL‐33 in the cell pellets and supernatants were analyzed.

### 5.23 Quantitative Real‐Time PCR

Total RNA was extracted using TRIzol reagent. The extracted RNA was then reverse transcribed into cDNA using a TIANGEN reverse transcription kit, and quantitative real‐time PCR was performed using TIANGEN SuperReal PreMix Plus (SYBR Green). The cycle threshold (Ct) values of qPCR samples were determined using the Q6 Real Time PCR Detection System. ΔCt was calculated by subtracting the Ct value of the internal control gene β‐actin from that of the target gene. The data were then normalized to the mean 2^‐ΔCt value of the control group.

### 5.24 IL‐33 Neutralization

Pregnant mice given normal drinking water or treated with DSS were administered IL‐33 neutralizing antibody or isotype IgG (2 mg/kg) via i.v. at E13.5.

### 5.25 Image Quantification

Immunofluorescence data were quantified using Leica LAS X software. Western blot images were quantified using LI‐COR Image Studio 5.0. For immunofluorescence analysis, laser power and gain were kept identical across samples during image acquisition. The fluorescence intensity of a region lacking the target protein was defined as the background (e.g., Tuj1 is not expressed in the VZ region; therefore, the VZ intensity was used as background). The true fluorescence intensity was obtained by subtracting the background fluorescence. For positive‐cell counting, a threshold was set such that the negative region contained no positive signal, and the number of positive cells was then counted. For western blotting, an area near the same lane without any band was used as the background; the true gray value was obtained by subtracting the background intensity.

### 5.26 Statistical Analysis

Statistical analyses were performed using GraphPad Prism 8.0. For immunoblotting, qPCR, and fluorescence intensity data, preprocessing was performed by normalization to the control mean: values in the control group were divided by the control‐group mean, and values in the treatment group were divided by the control‐group mean to obtain standardized values. The data used in this manuscript were first tested for normality and homogeneity of variance. If the groups met the assumptions of normal distribution and equal variances, two‐tailed unpaired *t*‐tests were used for two‐group comparisons, and one‐way ANOVA was used for comparisons involving three or more groups, and pairwise comparisons between groups were performed using Tukey's multiple comparisons test. If the data did not meet the assumptions of normality or homogeneity of variance between groups, the Mann–Whitney test or Dunn's multiple comparisons test was used. For multifactorial analyses, the normality and homogeneity of variances of the residuals were assessed before two‐way ANOVA was used. Specific statistical parameters for each experiment are detailed in the corresponding Figure legends. Data are shown as the mean ± SEM. All *P* values indicating statistical significance are labeled in the figure panels.

## Author Contributions

H.J. performed the experiments and drafted the manuscript. S.Z. provided the necessary technical support. K.M. and J.Z. jointly participated in the operation of some genotype identification experiments. Additionally, K.M. offered critical opinions on the revision of the manuscript. J.J. supervised the project and provided key guidance.

## Funding

This work was supported by grants from the National Key R&D Program of China (grant nos. 2024YFA1802202, 2025YFA1805100, 2024YFA1802600, 2024YFA1107500, 2023YFA1801500, and 2021YFA1101402); the National Natural Science Foundation of China (grant nos. 32521008, 32230040, 92368203, 32450088, 92468302, 32501026, 32300949, and 32300671); the Key Laboratory of Organ Regeneration and Reconstruction, Chinese Academy of Sciences (grant no. 2024KF04); the Initiative Scientific Research Program, Beijing Institute for Stem Cell and Regenerative Medicine (2025BS107); and the Initiative Scientific Research Program, Institute of Zoology, Chinese Academy of Sciences (grant nos. 2024IOZ0104 and 2023IOZ0304).

## Conflicts of Interest

The authors declare no conflict of interest.

## Supporting information




**Supporting File**: advs75298‐sup‐0001‐SuppMat.docx.

## Data Availability

The released data are available in NCBI GEO under accession numbers GSE310969 and GSE310970.
