# Supplementary material for: Gasdermin D‐Mediated Release of IL‐33 Results in Fetal Brain Developmental Abnormalities During Maternal Colitis
Source: Adv Sci (Weinh). 2026 Apr 13;13(38):e23784. doi: 10.1002/advs.202523784 (PMC13335094; doi:10.1002/advs.202523784)
Supplement: Supplementary file 1 — Supporting File: advs75298‐sup‐0001‐SuppMat.docx. [file ADVS-13-e23784-s001.docx]

**Supporting Information for**

Gasdermin D-mediated Release of IL-33 Results in Fetal Brain Developmental Abnormalities during Maternal Colitis

*Huiyang Jia**^1,2,3,5^, Shukui Zhang^1,2,3,5^, Kai Ma^1,2,3^, Jie Zhou^1,2,3^, Jianwei Jiao^1,2,3,4,6,*^*

**This file includes:**

Figures S1 to S8

Tables S1 to S4

**Figures S1–S8**


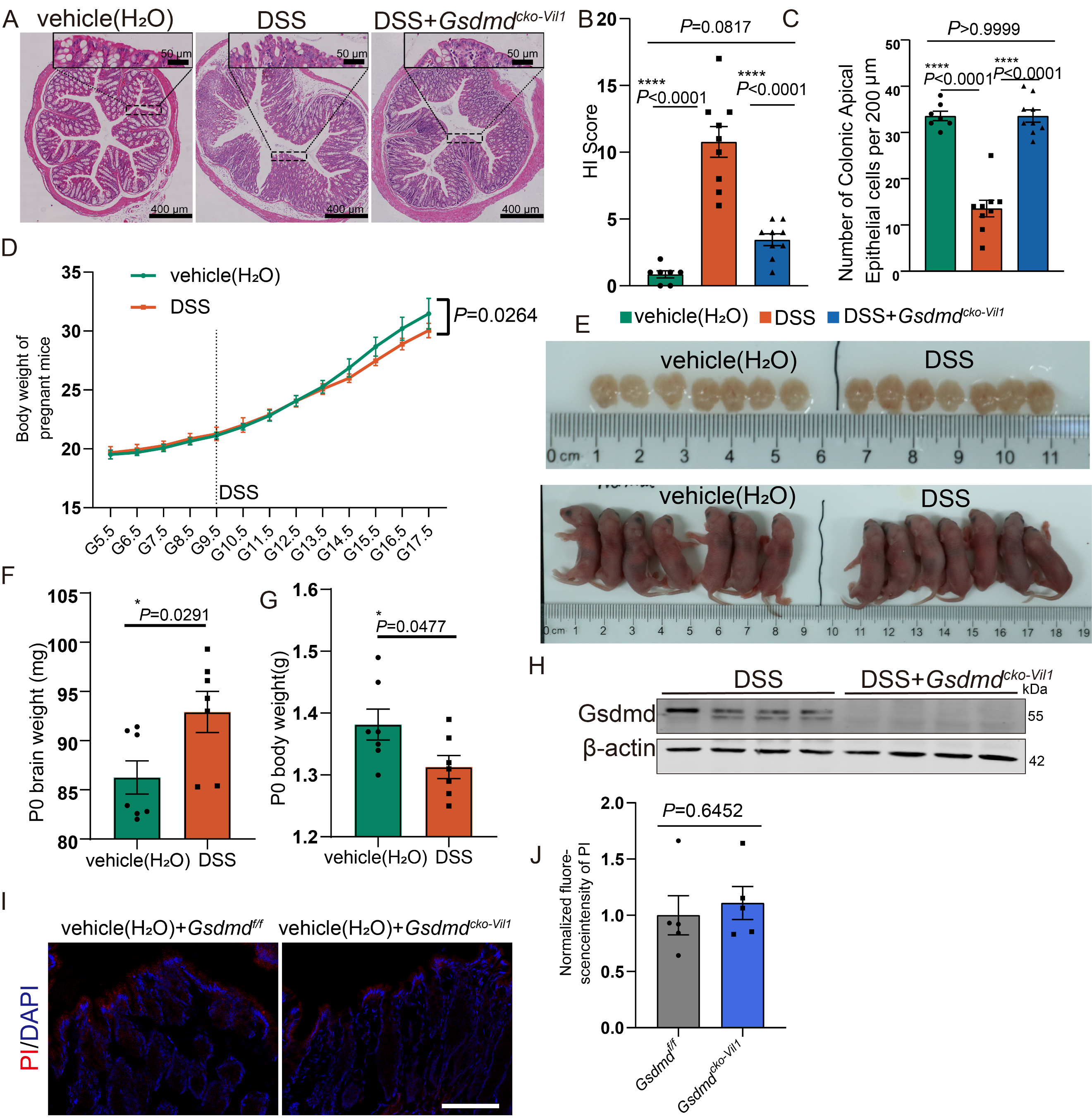


**Figure S1. Establishment of a maternal colitis model and its effects on brain weight and body weight in neonatal mice. Related to Figure 1.**

(**A**) HE staining reveals the pathological changes of colitis.

(**B**) Pathological score of colitis, related to (A). (n = 7, vehicle (H_2_O) group; n = 9, DSS group; n = 9, DSS+*Gsdmd^cko-Vil1^* group).

(**C**) Loss of colonic epithelial cells, related to (A). (n = 7, vehicle (H_2_O) group; n = 9, DSS group; n = 9, DSS+*Gsdmd^cko-Vil1^* group).

**(D)** Body weight growth curve of pregnant mice. (n = 4 per group).

(**E-G**) Photographs of whole-body and brain tissue of postnatal mice in the colitis group and the control group (E). Changes in brain weight (F) and body weight (G) of offspring mice from colitic mothers. (n = 7 per group).

(**H**) Western blot for detecting the knockout efficiency of *Gsdmd^cko-Vil1^* mice. (n = 4 per group).

(**I-J**) Representative image of PI-treated colon (I). Relative PI intensity in each group (J). (n = 5 per group). Scale bar = 100 μm.

Each data point in the plots represents one biological replicate. For experiments A, B, C, H, I and J, the number of technical replicates was 3. For experiments D-G, the number of technical replicates was 1. Data were analyzed with one-way ANOVA followed by Tukey's multiple comparisons test (B and C) and two-way repeated-measures ANOVA (D), and unpaired two-tailed t-tests (F, G and J). All data are presented as the mean ± SEM.


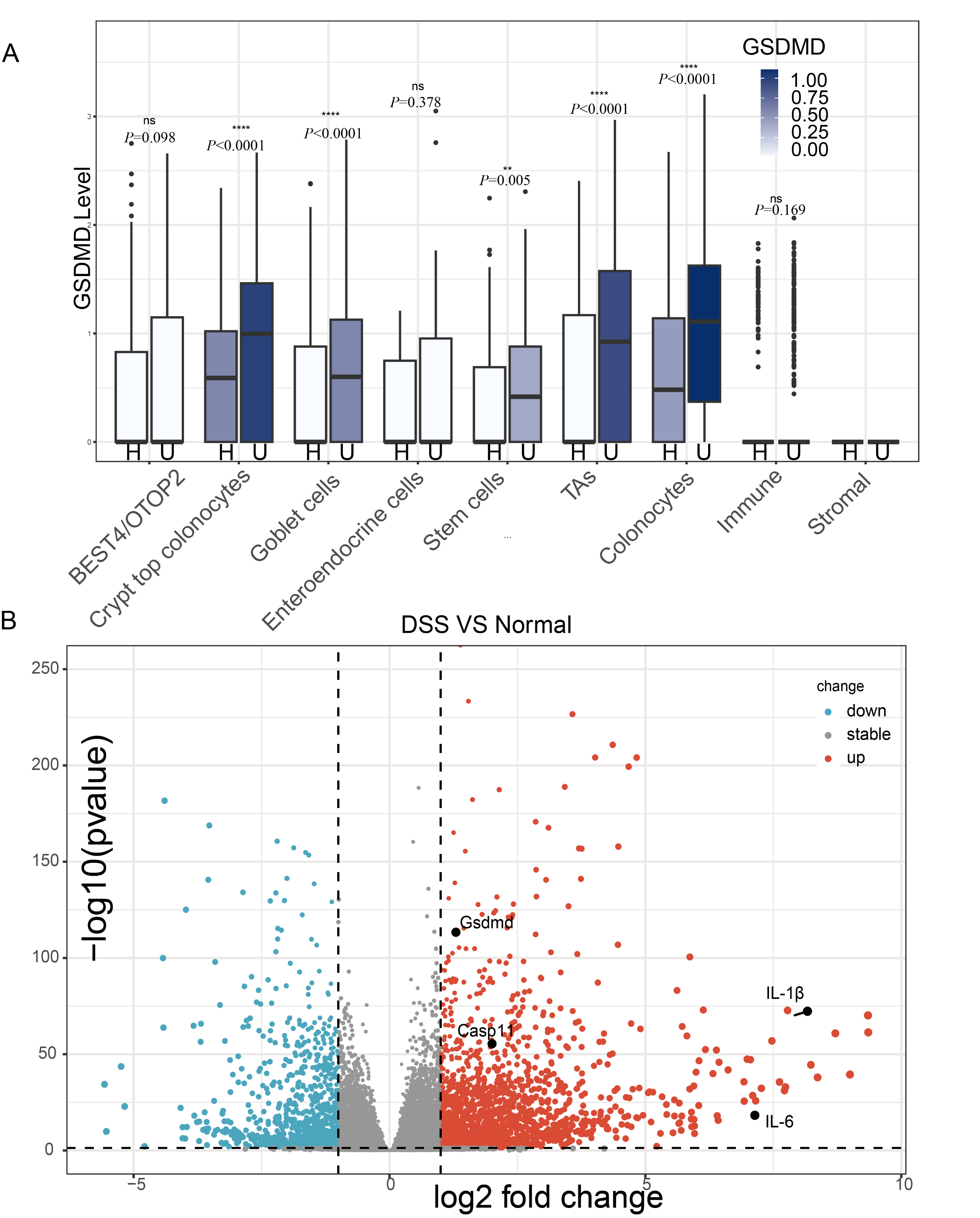


**Figure S2. The expression levels of GSDMD in various cell populations in human ulcerative colitis and the expression levels of pyroptosis-related genes in experimental colitis. Related to Figure 1.**

(**A**) The box plots show the expression levels of GSDMD in various cell populations in human normal colon (H: healthy) and ulcerative colitis (U: ulcerative colitis). The center line indicates the median, the box limits represent the 25th and 75th percentiles (IQR), the whiskers extend to the minimum and maximum values within 1.5 × IQR, and dots represent outliers. The color of the boxes indicates the average GSDMD expression level as shown in the color scale. (n = 3 per group).

(**B**) The volcano plot shows the changes in the expression levels of pyroptosis-related genes in the colonic tissue of mice with experimental colitis. (n = 4 per group).

For experiments A and B, the number of technical replicates was 1. Data were analyzed with the Wilcoxon rank-sum test (A). ns, not significant.


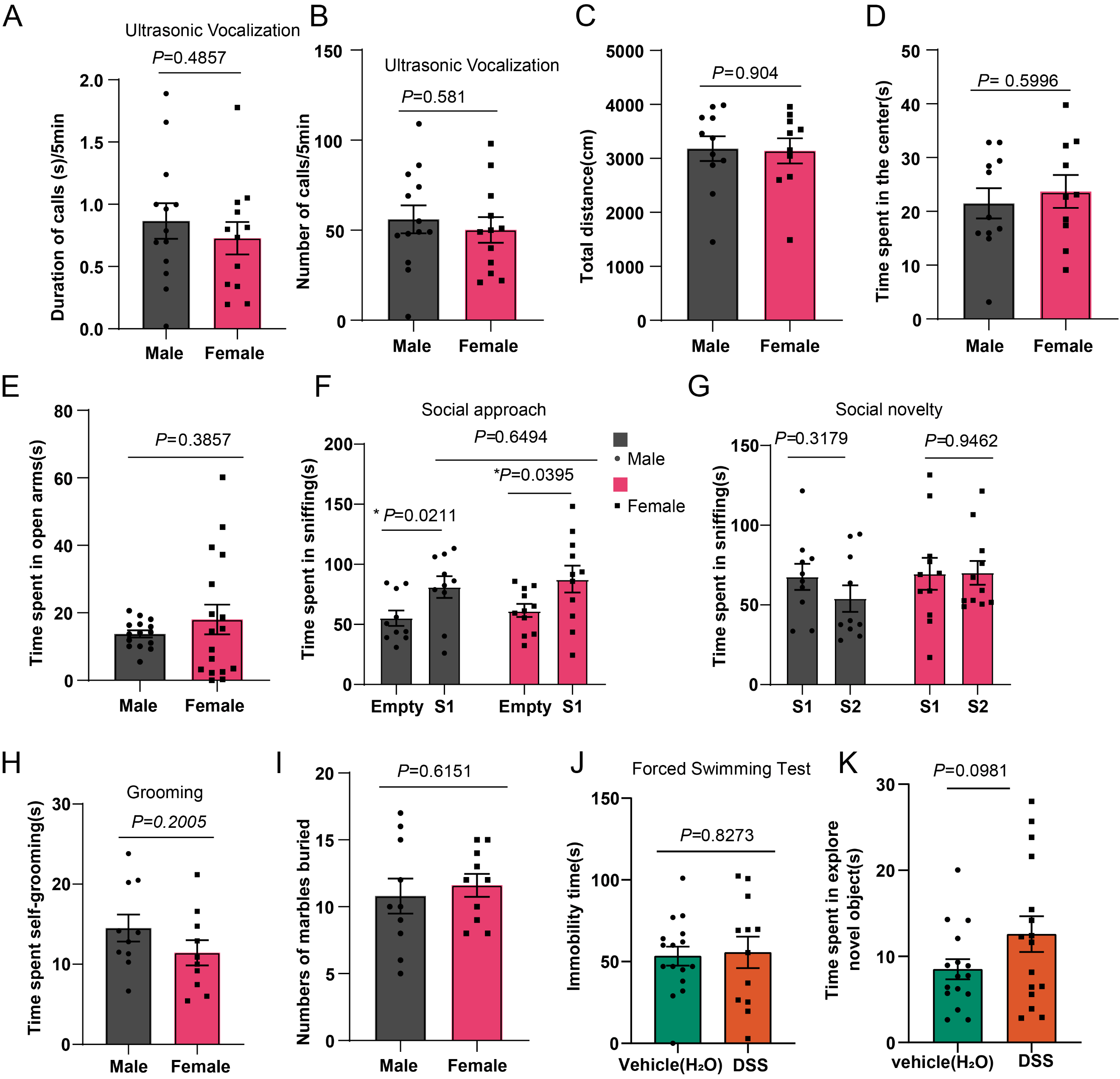


**Figure S3. Offspring behavioral abnormalities induced by DSS treatment are independent of sex, and DSS does not induce depressive-like behaviors or cognitive deficits. Related to Figure 2.**

(**A-B**) Duration (A) and number (B) of ultrasonic vocalizations (USVs) in male and female mice. (n = 13, male; n = 12, female).

(**C-D**) Total distance moved (C) and duration of exploration (D) in the central area in the open field. (n = 11, male; n = 10, female).

(**E**) Time spent in open arms. (n = 15, male; n = 17, female).

(**F)** Comparison of the durations of mice exploring Stranger 1 and the empty cage. (n = 10, male; n = 11, female).

(**G**) Comparison of the durations of mice exploring Stranger 1 and Stranger 2. (n = 10, male; n = 11, female).

(**H**) Duration of stereotypic grooming behavior in male and female mice within five minutes. (n = 10, male; n = 10, female).

(**I**) Number of marbles buried within 30 minutes. (n = 10, male; n = 10, female).

(**J**) Forced swim test: Duration of immobility in water in the control group and the DSS-treated offspring. (n = 16, vehicle (H_2_O) group; n = 12, DSS group).

(**K**) Duration of exploration of the novel object within five minutes. (n = 16 per group).

Each data point in the plots represents one biological replicate. The number of technical replicates was 1. All data were analyzed with unpaired two-tailed t-tests. All data are presented as the mean ± SEM.


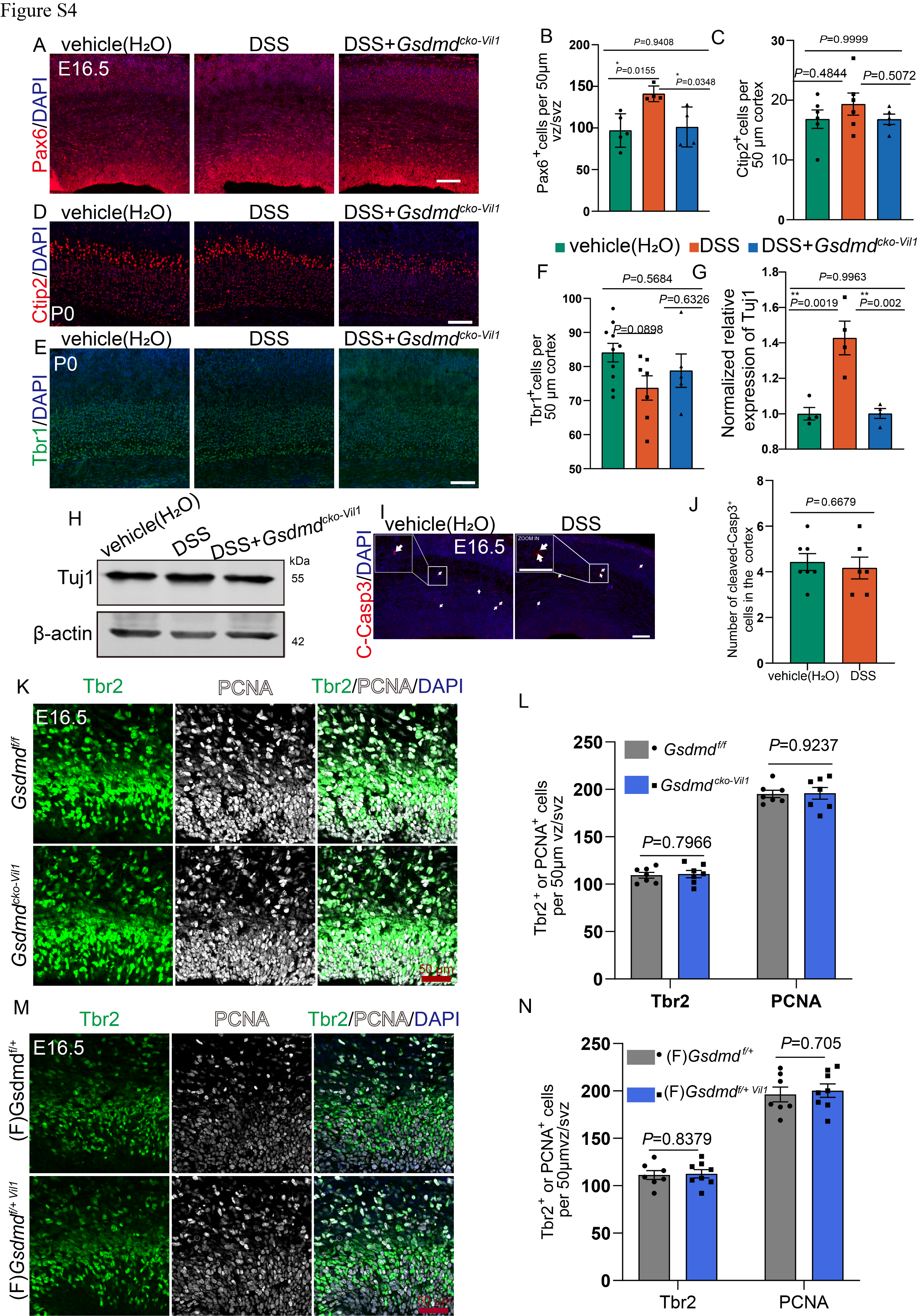


**Figure S4. Supplementary analysis of the proliferation, differentiation, apoptosis status of fetal cortex under the condition of colitis during pregnancy, and the proliferation and differentiation of neural stem cells in transgenic mice under normal drinking water conditions. Related to Figure 3.**

(**A-B**) Representative immunofluorescence images of Pax6^+^ cells in E16.5 cerebral cortex for each group (A), and bar graph showing the statistical analysis of Pax6^+^ cell number (B). (n = 5, vehicle (H_2_O) group; n = 4, DSS group; n = 4, DSS+*Gsdmd^cko-Vil1^* group). Scale bar = 100 μm.

(**C-D**) Representative immunofluorescence images of Ctip2^+^ cells at P0 (D), and statistical analysis of the number of Ctip2^+^ cells in the three groups of mice (C). (n = 6, vehicle (H_2_O) group; n = 6, DSS group; n = 5, DSS+*Gsdmd^cko-Vil1^* group). Scale bar = 100 μm.

(**E-F**) Representative immunofluorescence images of Tbr1^+^ cells at P0 (E), and statistical analysis of the number of Tbr1^+^ cells in three groups of mice (F). (n = 10, vehicle (H_2_O) group; n = 7, DSS group; n = 5, DSS+*Gsdmd^cko-Vil1^* group). Scale bar = 100 μm.

(**G-H**) Western blot analysis of the expression level of Tuj1 in the E16.5 cerebral cortex of three groups of mice (H), and normalized analysis of expression levels in each group (G). (n = 4 per group).

(**I-J**) Representative images showing the cortical distribution of apoptotic cells (white arrow) in the control group and DSS-treated group (I), and statistical analysis of the number of apoptotic cells (J). (n = 7, vehicle (H_2_O) group; n = 6, DSS group). Scale bar = 100 μm.

(**K-L**) Representative images (K) and statistical analysis graphs (L) of Tbr2 and PCNA in the fetal cerebral cortex of Gsdmd‑knockout (*Gsdmd^cko-Vil1^*) and WT (*Gsdmd^f/f^*) pregnant mice under normal drinking water conditions. (n = 7 per group). Scale bar = 50 μm.

(**M-N**) Representative images (M) and statistical analysis (N) of Tbr2 and PCNA in Gsdmd heterozygous conditional knockout ((F)*Gsdmd^f/+ Vil1^*) and wild-type ((F)*Gsdmd^f/+^*) fetal mice under normal drinking water conditions. (n = 7, (F)*Gsdmd^f/+^* group; n = 8, (F)*Gsdmd^f/+ Vil1^* group). Scale bar = 50 μm.

Each data point in the plots represents one biological replicate. All experiments had 3 technical replicates. Data were analyzed using one-way ANOVA followed by Tukey's multiple comparisons test (B, C, F and G) and unpaired two-tailed t-tests (J, L and N). All data are presented as the mean ± SEM.


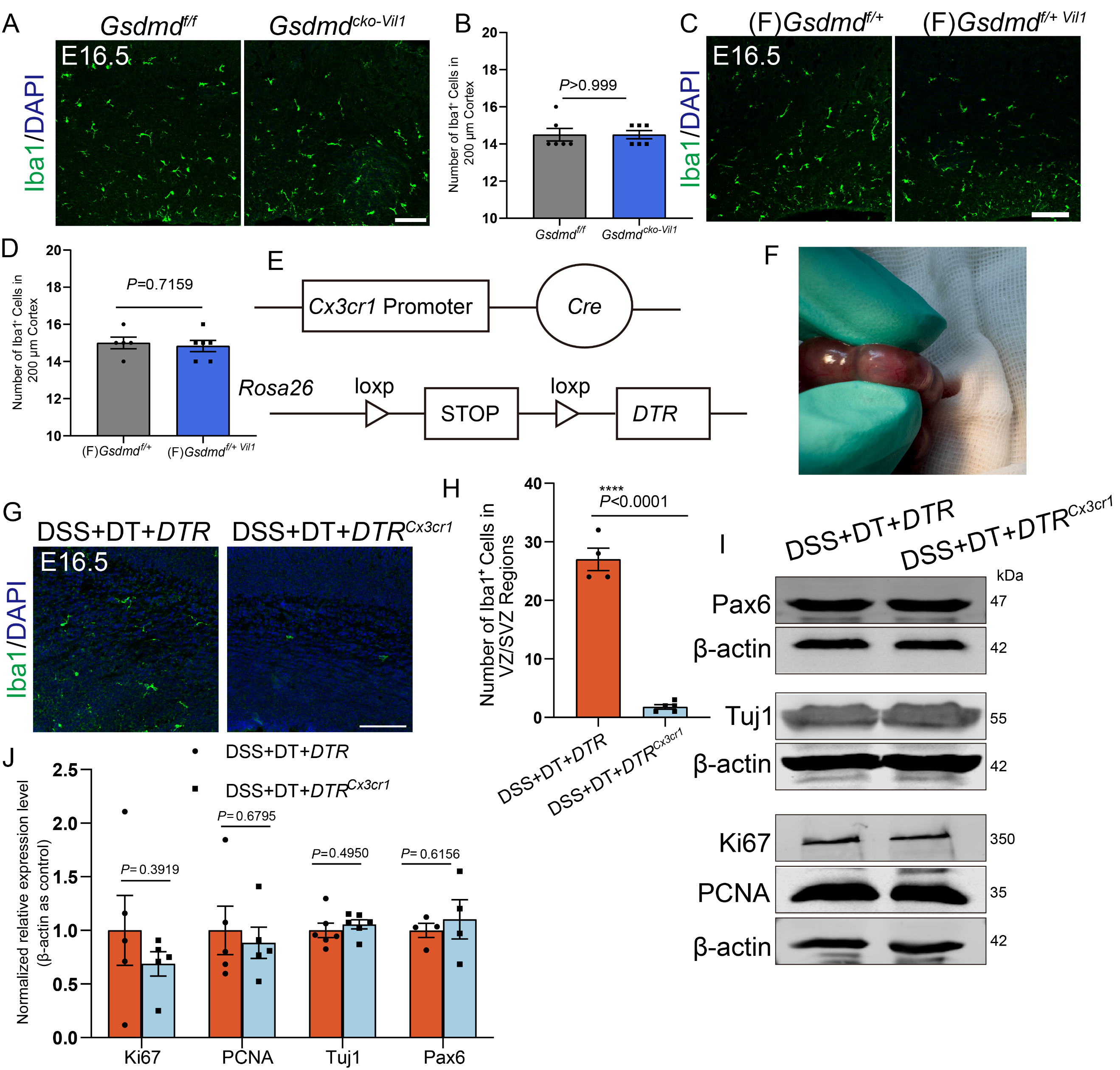


**Figure S5. The effect of maternal Gsdmd deficiency or fetal heterozygous knockout of Gsdmd on the number of microglia, and the effect of fetal microglia depletion via the DT-DTR method on cortical development in offspring of colitic dams. Related to Figure 4.**

**(A-B)** Representative images (A) and statistical analysis graphs (B) of Iba1 staining in the fetal cerebral cortex of Gsdmd‑knockout (*Gsdmd^cko-Vil1^*) and WT (*Gsdmd^f/f^*) pregnant mice under normal drinking water conditions. (n = 6 per group). Scale bar = 100 μm.

(**C-D**) Representative images (C) and statistical analysis (D) of Iba1 in Gsdmd heterozygous conditional knockout ((F)*Gsdmd^f/+ Vil1^*) and wild-type ((F)*Gsdmd^f/+^*) fetal mice under normal drinking water conditions. (n = 5, (F)*Gsdmd^f/+^* group; n = 6, (F)*Gsdmd^f/+ Vil1^* group). Scale bar = 100 μm.

(**E**) Construction of mice with microglia-specific expression of diphtheria toxin receptor (DTR).

(**F**) Intracerebroventricular injection of diphtheria toxin mixed with Fast Green.

(**G-H**) Depletion of microglia in the fetal cerebral cortex of maternal mice with colitis using diphtheria toxin (DT) (G), and statistical analysis of the depletion efficiency (H). (n = 4, *DTR* group; n = 5, *DTR^cx3cr1^* group). Scale bar = 100 μm.

(**I-J**) Representative immunoblot images of Pax6, Tuj1, PCNA, and Ki67 following the depletion of microglia (I), and statistical analysis of their expression levels (J). (Pax6, n = 4 per group; Tuj1, n = 6 per group; PCNA, n = 5 per group; Ki67, n = 5 per group).

Each data point in the plots represents one biological replicate. For experiments A, B, C, D, G, H, I and J, the number of technical replicates was 3. For experiments E-F, the number of technical replicates was 1. Data were analyzed with unpaired two-tailed t-tests (B, D, H and J). All data are presented as the mean ± SEM.


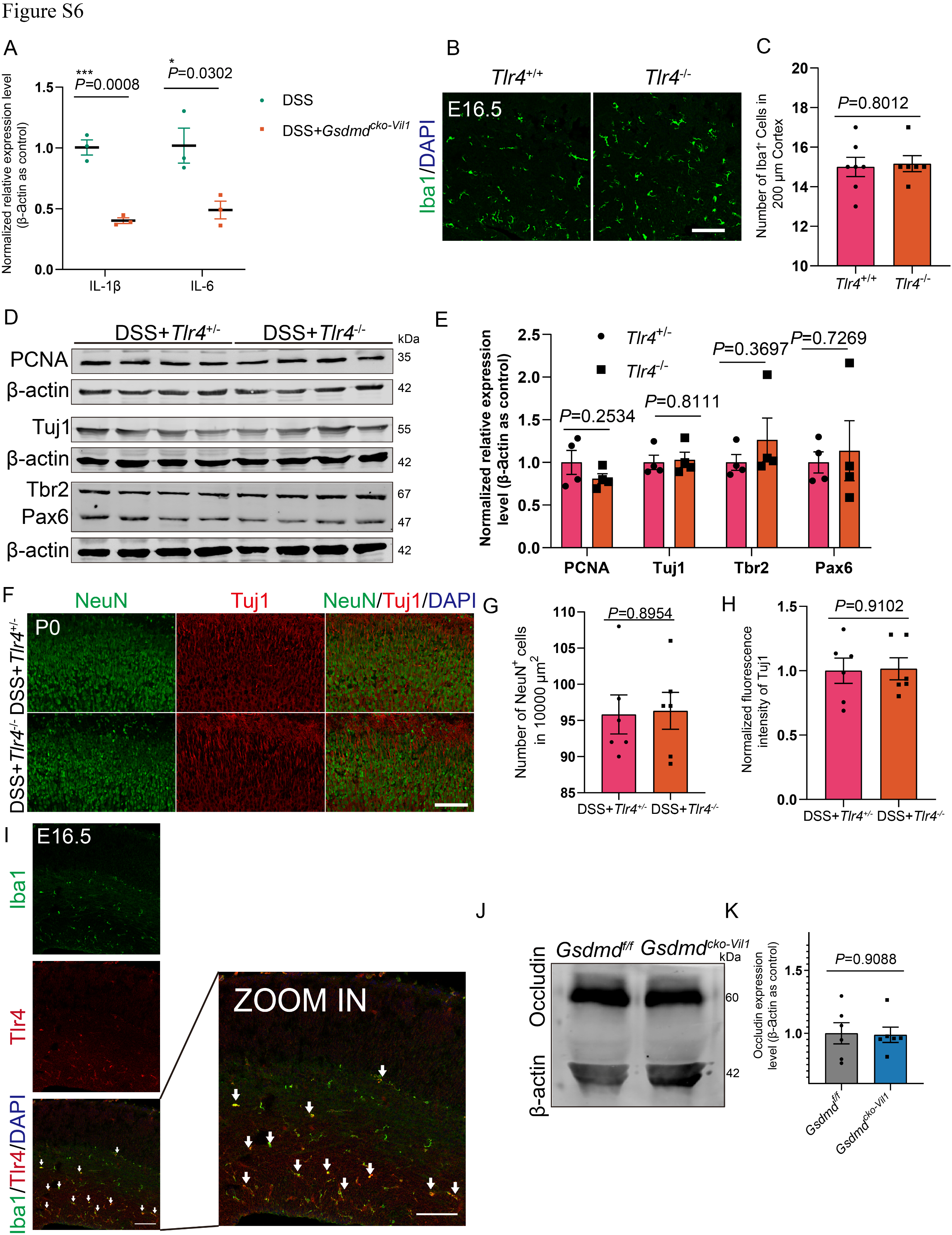


**Figure S6. Knockout of Gsdmd in pregnant mice attenuated the expression of Tlr4‑related genes in the fetal brain, Tlr4 is mainly expressed in microglia, and Tlr4 knockout does not affect NSCs, and** **knockout of Gsdmd in colonic epithelium has no effect on tight junctions. Related to Figure 5.**

(**A**) Detection of Tlr4 downstream genes in fetal cerebral cortex: DSS vs DSS + *Gsdmd^cko-Vil1^*. (n = 3 per group).

(**B-C**) Representative staining images (B) of Iba1 and corresponding statistical analysis (C) in *Tlr4* knockout and wild-type mice under normal drinking water conditions. (n = 7, *Tlr4^+/+^* group; n = 6, *Tlr4^-/-^* group). Scale bar = 100 μm.

(**D-E**) Western blot images (D) and statistical analysis of the expression levels (E) of PCNA, Tuj1, Tbr2, and Pax6 in the fetal cortex of pregnant mice with colitis. (n = 4 per group).

(**F-H**) Representative staining images (F) of the neuronal markers Tuj1 and NeuN and corresponding statistical analysis (G and H) in *Tlr4* knockout and heterozygous mice at postnatal day 0 (P0) under maternal colitis. (n = 6 per group). Scale bar = 100 μm.

(**I**) Immunofluorescence detection of the co-localization of Tlr4 and Iba1 (white arrow). Scale bar = 100 μm.

(**J-K**) Representative Western blotting images (J) and corresponding statistical analysis (K) of colonic Occludin in Gsdmd conditional knockout and wild‑type pregnant mice under normal drinking water conditions. (n = 6 per group).

Each data point in the plots represents one biological replicate. All experiments were performed with 3 technical replicates. Data were analyzed with unpaired two-tailed t-tests. All data are presented as the mean ± SEM.


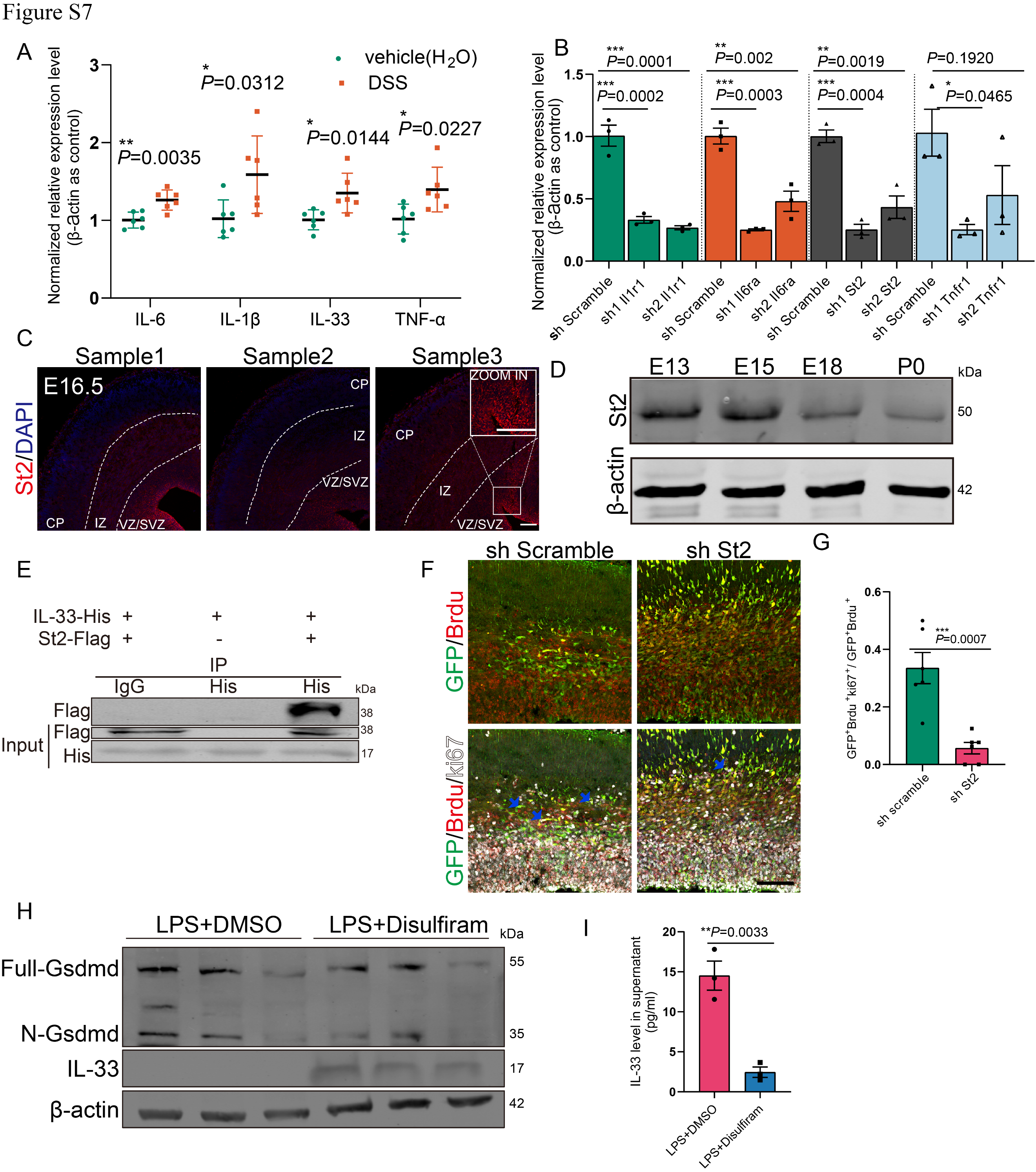


**Figure S7. DSS treatment of pregnant mice increased the expression of pyroptosis‑related cytokines; construction of the knockdown plasmids and the distribution and function of St2 in the cortex; IL‑33 can bind to St2, and inhibition of Gsdmd pore‑formation reduced the release of IL‑33. Related to Figure 6.**

(**A**) Relative expression levels of four pyroptosis‑related cytokines (determined by qPCR). (n = 6 per group).

(**B**) Detection of the knockdown efficiency of the plasmid by qPCR. (n = 3 per group).

(**C**) Immunofluorescence shows the distribution of St2 in the brain. Scale bar = 100 μm.

(**D**) Western blot images show the changes in St2 expression levels from E13 to P0.

(**E**) The binding between IL-33 and St2 was detected by co-immunoprecipitation (Co-IP) combined with Western blotting.

(**F-G**) Representative immunofluorescence images cell cycle maintenance (blue arrow) (F) and statistical analysis of the number of cells with cell cycle maintenance (G). (n = 6 per group). Scale bar = 100 μm.

(**H-I**) Effect of disulfiram on the release of IL-33 from colonic epithelial cells under LPS stimulation. Intracellular IL-33 levels were detected by Western blotting (H), and the concentration of IL-33 in the cell supernatant was determined by ELISA (I).

Each data point in the plots represents one biological replicate. All experiments were performed with 3 technical replicates. Data were analyzed with unpaired two-tailed t-tests (A, B, G and I). All data are presented as the mean ± SEM.


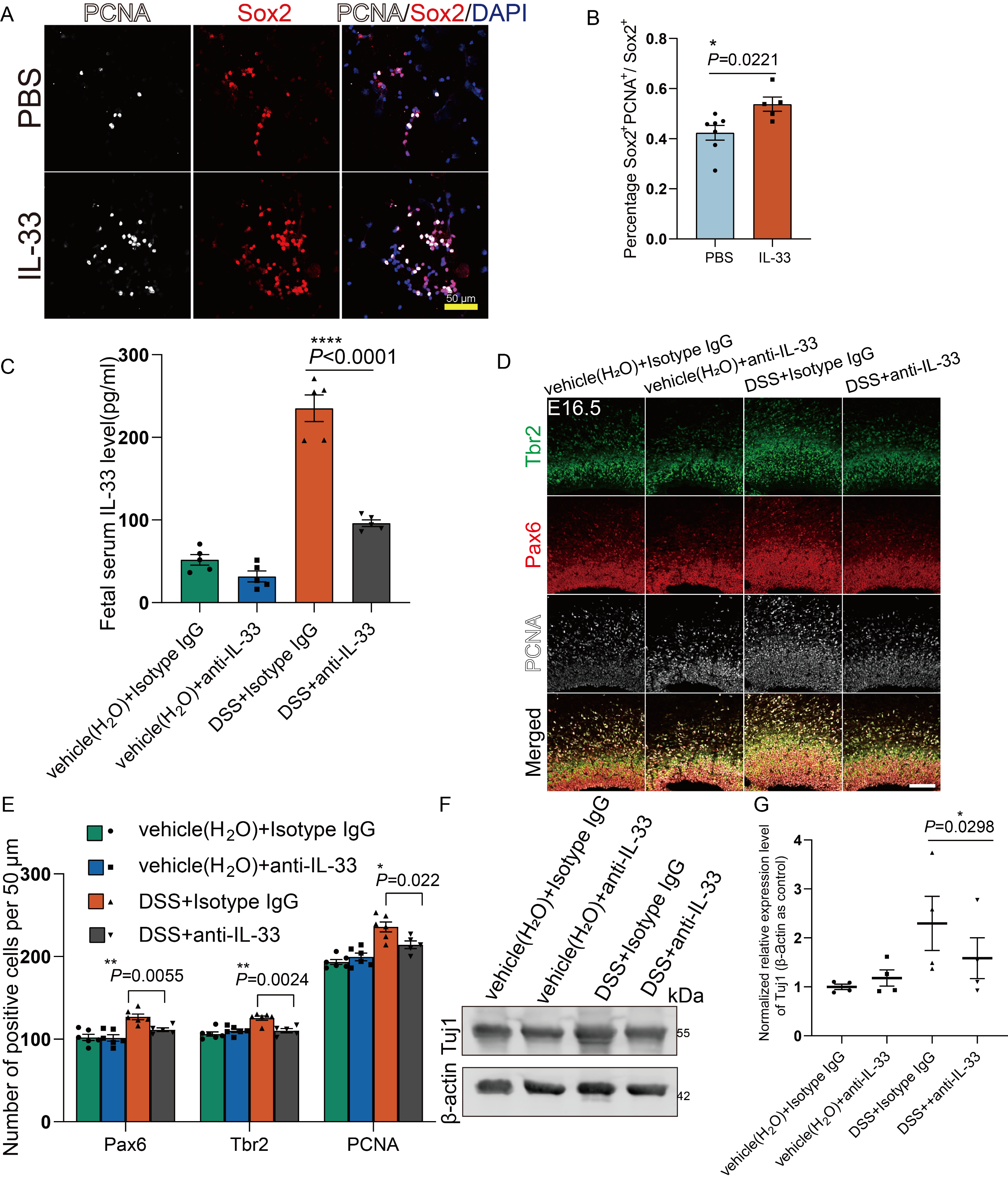
**Figure S8. IL-33 regulates the proliferation of neural stem cells;** **neutralization of IL‑33 can rescue the abnormalities in neurogenesis caused by colitis in pregnant mice. Related to Figure 7.**

(**A-B**) Representative images (A) and statistical analysis (B) of the expression status of PCNA and Sox2 in cultured neural stem cells treated with IL‑33 in vitro. (n = 7, PBS group; n = 5, IL-33 group). Scale bar = 50 μm.

(**C**) The serum concentration of IL‑33 was detected by ELISA after the addition of a neutralizing antibody or isotype IgG. (n = 5 per group).

(**D-E**) Representative images (D) and statistical analysis (E) of Pax6, Tbr2 and PCNA after treatment with a neutralizing antibody. (n = 5, DSS+anti-IL-33 group; n = 6 for each remaining group). Scale bar = 100 μm.

(**F-G**) Representative Western blot images (F) of Tuj1 and the corresponding statistical results (G). (n = 4 per group).

Each data point in the plots represents one biological replicate. All experiments were performed with 3 technical replicates. Data were analyzed with unpaired two-tailed t-tests (B, C, E and G). All data are presented as the mean ± SEM.

**Table S1-S4**

**Table S1. Short hairpin RNA (shRNA) sequence**

| sh RNA | Sequences (5’-3’) |
| --- | --- |
| sh1 St2 | CAAGCTGCAATATCCCTGATT |
| sh2 St2 | GTGGTTTAAGAACTGCAAAGC |
| sh1 Tnfr1 | TCAGTTGCAAGACATGTCGGA |
| sh2 Tnfr1 | AGAAAGTGAGTGCGTCCCTTG |
| sh1 Il1r1 | CGACACCATAATTTGGTACAA |
| sh2 Il1r1 | CCATTGTCTAAACACCGCTTA |
| sh1 Il6ra | CGAGGATCAGTACGAAAGTTC |
| sh2 Il6ra | GATACCGACCTGTATGGTCAA |
| sh scramble | CCTAAGGTTAAGTCGCCCTCG |

**Table S2. qPCR primers**

| Target | Sequences (5’-3’) |
| --- | --- |
| qPCR-St2 | F: TCAGAAGCCCCAACTTGAATA  R: CCTTGGCTCTTGGAGAGCTT |
| qPCR1-Tnfr1 | F: TACTTGGTGAGTGACTGTCCGA  R: CTTGTCAGCTTGGCAAGGAG |
| qPCR2-Tnfr1 | F: TTCAACGGCACCGTGACAATC  R: GAGGTAGGCACAACTTCATA |
| qPCR1- Il1r1 | F: GCCTCTGCTGTCGCTGGAGA  R: GGAGTCCCGGTCCGCTGATAT |
| qPCR2- Il1r1 | F: GATACCAGATGCCAGCCCAAC  R: GGATCCAGGGTTAGTAAGCG |
| qPCR1-Il6ra | F: CCTTGGATAGAGCCCAGGACC  R: CCTCCAGCTACCAGGAATGTG |
| qPCR-IL-33 | F: TAGGAAAGAACCCACGAAA  R: TGTCAACAGACGCAGCAAA |
| qPCR-IL-1β | F: AAGAAGAGCCCATCCTCTGTG  R: GGAGCCTGTAGTGCAGTTGTCTA |
| qPCR-IL-6 | F: TCTTGGGACTGATGCTGGTG  R: TTGGGAGTGGTATCCTCTGTGAA |
| qPCR-TNF-α | F: CGACGTGGAACTGGCAGAA  R: AGACAGAAGAGCGTGGTGGC |
| qPCR-β-actin | F: CAGCCTTCCTTCTTGGGTAT  R: GGCATAGAGGTCTTTACGG |

**Table S3. Marker genes used for cell clustering**

| **Cell type** | **Marker genes** |
| --- | --- |
| BEST4/OTOP2 | OTOP2, BEST4 |
| Crypt top colonocytes | AQP8, CEACAM1 |
| Goblet cells | MUC2, ITLN1 |
| Enteroendocrine cells | CHGA, NEUROD1 |
| Stem cells | LGR5, RGMB, SMOC2, ASCL2 |
| TAs | MKI67, KI67, PCNA, TOP2A, CCNA2, MCM5 |
| Colonocytes | CA1 |
| Immune | CD45, PTPRC |
| Stromal | CD10, MME, PECAM1 |

**Table S4 Antibodies and reagents**

| **Antibody** | **Company** | **Source** | **Usage** |
| --- | --- | --- | --- |
| Rabbit anti-Tuj1 | Sigma | Cat# T2200; RRID: AB_262133 | 1:1000 |
| Rabbit anti-Tbr1 | Abcam | Cat# ab31940; RRID: AB_2200219 | 1:1000 |
| Rat anti-Ctip2 | Abcam | Cat# ab18465; RRID: AB_2064130 | 1:1000 |
| Mouse anti-Satb2 | Abcam | Cat# ab51502; RRID: AB_882455 | 1:100 |
| Rabbit anti-Cux1 | Proteintech | Cat# 11733-1-AP; RRID: AB_2086995 | 1:1000 |
| Rabbit anti-Pax6 | Millipore | Cat# AB2237; RRID: AB_1587367 | 1:1000 |
| Rabbit anti-Pax6 | Proteintech | Cat# 12323-1-AP; RRID: AB_2159695 | 1:1000 |
| Rat anti-Tbr2 | Invitrogen | Cat# 14-4875-82; RRID: AB_11042577 | 1:1000 |
| Rabbit anti-Tbr2 | Proteintech | Cat# 83945-5-RR; RRID: AB_3671525 | 1:5000 |
| Rabbit anti-Tbr2 | Abcam | Cat# ab23345; RRID: AB_778267 | 1:1000 |
| Rabbit anti-PCNA | Santa Cruz | Cat# sc-7907; RRID: AB_2160375 | 1:200 |
| Rabbit anti-Ki67 | Abcam | Cat# ab15580; RRID: AB_443209 | 1:1000 |
| Rat anti-Brdu | Abcam | Cat# ab6326; RRID: AB_305426 | 1:1000 |
| Rabbit anti-Cd68 | Proteintech | Cat# 28058-1-AP; RRID: AB_2881049 | 1:1000 |
| Rabbit anti-Iba1 | Wako | Cat# 019-19741; RRID: AB_839504 | 1:1000 |
| Rabbit anti-GSDMA | Abcam | Cat# ab232254; RRID: AB_3608289 | 1:1000 |
| Rabbit anti-GSDMC2+GSDMC3 | Abcam | Cat# ab229896; RRID: AB_2938769 | 1:1000 |
| Rabbit anti-GSDME | Abmart | Cat# P79886; RRID: AB_3714554 | 1:1000 |
| Rabbit anti-GSDMD | Abcam | Cat# ab209845; RRID: AB_2783550 | 1:1000 |
| Rabbit anti-GSDMD | Proteintech | Cat# 20770-1-AP; RRID: AB_10696319 | 1:1000 |
| Rabbit anti-Caspase-11 | Abcam | Cat# ab180673; RRID: AB_2923217 | 1:1000 |
| Mouse anti-Tlr4 | Proteintech | Cat# 66350-1-Ig; RRID: AB_2881730 | 1:1000 |
| CD326 (EpCAM) Antibody (G8.8), FITC | Thermo Fisher | Cat# 11-5791-82; RRID: AB_11151709 | 1 μl:10^5^ cells |
| CD45-PE Antibody | Invitrogen | Cat# 12-0451-82  RRID: AB_465668 | 1 μl:10^5^ cells |
| Rat anti-Cd326(EpCAM) | Thermo Fisher | Cat# 14-5791-81; RRID: AB_953624 | 1:1000 |
| Rabbit anti-Zo-1 | Thermo Fisher | Cat# 40-2200; RRID: AB_2533456 | 1:1000 |
| Rabbit anti-Occludin | Proteintech | Cat# 27260-1-AP; RRID: AB_2880820 | 1:1000 |
| Mouse anti-Beta Actin | Proteintech | Cat# 66009-1-Ig; RRID: AB_2687938 | 1:1000 |
| Rabbit anti-St2 | Proteintech | Cat# 11920-1-AP; RRID: AB_906359 | 1:1000 |
| Mouse anti-IL-33 | Proteintech | Cat# 12372-1-AP; RRID: AB_2877852 | 1:1000 |
| Mouse anti-GFP | ABclonal | Cat# AE012; RRID: AB_2770402 | 1:1000 |
| Mouse anti-His-tag | Proteintech | Cat# 66005-1-Ig; RRID: AB_11232599 | 1:1000 |
| Rabbit anti-Beta Actin | Proteintech | Cat# 20536-1-AP; RRID: AB_10700003 | 1:60000 |
| IL-33 neutralizing antibody | Adipogen | Cat# AG-27B-0013PF; AB_2490504 | 2 mg/kg |
| IRDye 680LT Donkey anti-Rabbit IgG | LI-COR | Cat# 926-68023; RRID: AB_10706167 | 1:10000 |
| IRDye 800CW Donkey anti-Mouse IgG | LI-COR | Cat# 926-32212; RRID: AB_621847 | 1:10000 |
| IRDye 800CW Donkey anti-Goat IgG | LI-COR | Cat# 925-32214; RRID: AB_2687553 | 1:10000 |
| IRDye 800CW Goat anti-Rat IgG | LI-COR | Cat# 925-32219; RRID: AB_2721932 | 1:10000 |
| Cy3-conjugated Anti-Mouse IgG | Jackson | Cat# 715-165-150; RRID: AB_2340813 | 1:1000 |
| Cy5-conjugated Anti-Mouse IgG | Jackson | Cat# 715-175-151; RRID: AB_2340820 | 1:1000 |
| Alexa Fluor 488-conjugated Anti-Rabbit IgG | Jackson | Cat# 711-546-152; RRID: AB_2340619 | 1:1000 |
| Cy3-conjugated Anti-Rabbit IgG | Jackson | Cat# 711-165-152; RRID: AB_2307443 | 1:1000 |
| Cy5-conjugated Anti-Rabbit IgG | Jackson | Cat# 711-175-152; RRID: AB_2340607 | 1:1000 |
| Alexa Fluor 488-conjugated Anti-Rat IgG | Jackson | Cat# 712-545-150; RRID: AB_2340683 | 1:1000 |
| Cy3-conjugated Anti-Rat IgG | Jackson | Cat# 712-165-150; RRID: AB_2340666 | 1:1000 |
| Cy5-conjugated Anti-Rat IgG | Jackson | Cat# 712-175-150; RRID: AB_2340671 | 1:1000 |
| **Chemicals, peptides, and recombinant proteins** |  |  | Usage |
| Dextran Sulfate sodium (DSS) | MP Biomedicals | Cat# 0216011080 | 2.5% |
| Paraformaldehyde | Sigma | Cat# 158127-500G | 4% |
| Lipopolysaccharide (LPS) | Sigma | Cat# L4391 | 1 μg/ml |
| Propidium iodide | Sigma | Cat# P4170 | 1 00 μg/ml |
| Brdu | Sigma | Cat# B5002-1G | 10 mg/ml |
| DAPI | Sigma | Cat# D9542-10MG | 1 μg/ml |
| Fast Green | Sigma | Cat# F7258 | N/A |
| TRIzol | Ambion Life Technology | Cat# 15596018 | N/A |
| IL-33 Protein | NOVOPROTEIN | Cat# CG73 | 5 ng/ml |
| IL-33 His-tag | MCE | Cat# HY-P700208AF | 100 μg/ml |
| LPS-EB- Biotin | Invivogen | Cat# NC1838287 | 500 μg/ml |
| DMEM | Gibco | Cat# 11995-065 | N/A |
| DMEM/F12(1:1) | Gibco | Cat# 11330-032 | N/A |
| Neurobasal Medium | Gibco | Cat# 21103-049 | N/A |
| B-27 Supplement without vitamin A | Gibco | Cat# 12587-010 | N/A |
| FBS | Gibco | Cat# 16000044 | 10% |
| Penicillin/Streptomycin | Thermo Fisher | Cat# 15070063 | 1% |
| 2,2,2- tribromoethanol | Sigma | Cat# T48402 | N/A |
| **Experimental models: Cell lines** |  |  |  |
| Neuro-2a | ATCC | RRID: CVCL_0470 | N/A |
| **Experimental models: Organisms/Strains** |  |  |  |
| Mouse: Gsdmd^fl/fl^ | RIKEN BRC | Stock No: RBRC10762 | N/A |
| Mouse: Vil1-Cre | Jackson Laboratory | Stock No: 021504 | N/A |
| Mouse: Cx3cr1-Cre | Jackson Laboratory | Stock No: 025524 | N/A |
| Mouse: Rosa26-iDTR | Cyagen | Stock No: C001477 | N/A |
| Mouse: Tlr4-KO | Cyagen | Stock No: C001234 | N/A |
